# Role for *N*-glycans and calnexin-calreticulin chaperones in SARS-CoV-2 Spike maturation and viral infectivity

**DOI:** 10.1126/sciadv.abq8678

**Published:** 2022-09-23

**Authors:** Qi Yang, Anju Kelkar, Anirudh Sriram, Ryoma Hombu, Thomas A. Hughes, Sriram Neelamegham

**Affiliations:** ^1^ Chemical and Biological Engineering, State University of New York, Buffalo, NY 14260, USA.; ^2^ Biomedical Engineering, State University of New York, Buffalo, NY 14260, USA.; ^3^ Medicine, State University of New York, Buffalo, NY 14260, USA.; ^4^ Clinical and Translational Research Center.; ^5^ Cell, Gene and Tissue Engineering Center, Buffalo 14260, NY, USA.

## Abstract

Functional and epidemiological data suggest that *N*-linked glycans on the SARS-CoV-2 Spike protein may contribute to viral infectivity. To investigate this, we created a panel of N-to-Q mutations at *N*-glycosylation sites proximal to the Spike S1-S2 (N61, N603, N657, and N616) and S2′ (N603 and N801) proteolysis sites. Some of these mutations, particularly N61Q and N801Q, reduced Spike incorporation into Spike-pseudotyped lentivirus and authentic SARS-CoV-2 virus-like particles (VLPs). These mutations also reduced pseudovirus and VLP entry into ACE2-expressing cells by 80 to 90%. In contrast, glycan mutations had a relatively minor effect on cell surface expression of Spike, ACE2 binding, and syncytia formation. A similar dichotomy in function was observed when virus was produced in host cells lacking ER chaperones, calnexin and calreticulin. Here, while both chaperones regulated pseudovirus function, only VLPs produced in calnexin KOs were less infectious. Overall, Spike *N*-glycans are likely critical for SARS-CoV-2 function and could serve as drug targets for COVID-19.

## Teaser

*N*-linked glycans on Spike protein and the host lectin-binding chaperone calnexin play a critical role in SARS-CoV-2 infection.

## INTRODUCTION

Vaccines have emerged as a key public health measure to mitigate severe acute respiratory syndrome coronavirus 2 (SARS-CoV-2) infection and coronavirus disease-2019 (COVID-19). These vaccines commonly target the trimeric glycoprotein called “Spike,” as this is ubiquitously expressed in virions and required for viral entry into host cells. However, there is grave risk that the virus will continue to mutate to evade host immunity and thus vaccine efficacy will wane. This is exemplified by the emergence of variants that exhibit substantial immune escape including B.1.617.2 (Delta), B.1.1.529 (Omicron) and additional descendent lineages (*1*, *2*). In this regard, several mutations in the Spike receptor-binding domain (RBD) are well tolerated and some of these modifications even enhance Spike binding to its primary cell entry receptor, angiotensin-converting enzyme-2 (ACE2) (*3*, *4*). Thus, there is an urgent need to identify aspects of viral biology that are critical for infectivity and to develop interventions targeting these vulnerabilities. Given that glycans are an underleveraged target in the field of virology, this study examines the mechanism(s) by which these complex carbohydrates regulate virus function.

The Spike protein contains 22 potential *N*-glycosylation sites that are mostly occupied and additional sites for *O*-linked glycosylation (*5*–*7*). These glycans can contribute to the immunological shield, as Spike deglycosylation enhances immune response (*8*). In addition, we have previously reported that both the *N*- and *O*-linked glycans are likely to contribute to efficient viral entry into human cells that express ACE2, with *N*-glycans playing a more dominant role (*9*). These observations were made by generating Spike protein pseudotyped lentivirus (“pseudovirus”) lacking extended *N*-glycans because of their production in 293T cells devoid of GlcNAcT1/*MGAT1* activity and by the synthesis of *O*-glycan–deficient pseudovirions from core-1 GalT1/*C1GALT1* knockout (KO) cells. Supporting the importance of *N*-glycans in Spike function, Li *et al.* (*10*) observed that pseudoviral infectivity in a variety of cell types was generally reduced upon implementing site-specific mutations that ablated selected *N*-glycans. In addition, recent evidence suggests that aside from its major ligand ACE2, Spike *N*-glycans may also directly bind a variety of C-type and Tweety family member 2 lectins (*11*, *12*). It is also reported that the Spike protein RBD contains a positively charged interface proximal to the ACE2 binding site that potentially binds both heparan sulfate glycosaminoglycans (*13*–*15*) and monosialylated glycolipids (*16*). Last, small-molecule inhibitors of *N*-glycan processing, mostly iminosugars, have been shown to reduce viral infectivity (*17*, *18*).

The 22 *N*-linked glycans of Spike may be classified into four groups on the basis of their location in the three-dimensional prefusion crystal structure: (i) Eight glycans from N17-N282 that lie within the N-terminal domain (NTD); (ii) glycans N331 and N343 at the base of the RBD, which may either act as direct binding partners or regulate the up-down conformation of this critical receptor binding epitope (*19*); (iii) glycans at N61, N603, N616, N657, and N801, which lie proximal to either the S1-S2 or S2′ proteolysis site that are critical for viral fusion and entry. The possibility that these glycans affect viral fusion during entry and/or syncytia formation remains unexamined (*20*, *21*); and (iv) the remaining carbohydrates at N709, N717, and N1074 to 1194 that lie closer to the base of the intact Spike protein. In the postfusion structure, five of these glycans (N1098, N1134, N1158, N1173, and N1194) are uniformly spaced at ~4-nm distance along the long axis of the S2 trimer (*22*). On the basis of molecular modeling, it is predicted that some of these carbohydrates may affect the rate of virus-host membrane fusion during entry (*23*).

To investigate the roles of these glycans, the current manuscript examined the function of the carbohydrates located proximal to the S1-S2 and S2′ proteolysis site. To this end, a series of site-specific single, double, and triple *N*-glycosylation site mutations [Asn(N) to Gln(Q)] were introduced into Spike. Several of these glycan mutations partially reduced Spike incorporation into pseudovirus and SARS-CoV-2 virus-like particles (VLP), particularly the N61Q and N801Q mutations. These mutations also severely impaired viral entry, although their impact on Spike cell surface expression, ACE2 binding function, and syncytia formation was small. A similar dichotomy was observed when pseudovirus and SARS-CoV-2 VLPs were produced in CRISPR-Cas9 KOs lacking the glycoprotein folding chaperones calnexin (*CANX*) and calreticulin (*CALR*). Overall, the study suggests that beyond acting as an immunological shield or direct receptor binding partner, *N*-glycans on SARS-CoV-2 Spike protein may have additional, essential roles in regulating viral infectivity.

## RESULTS

### *N*-glycans are essential for SARS-CoV-2 function in pseudovirus model

**Fig. 1.**
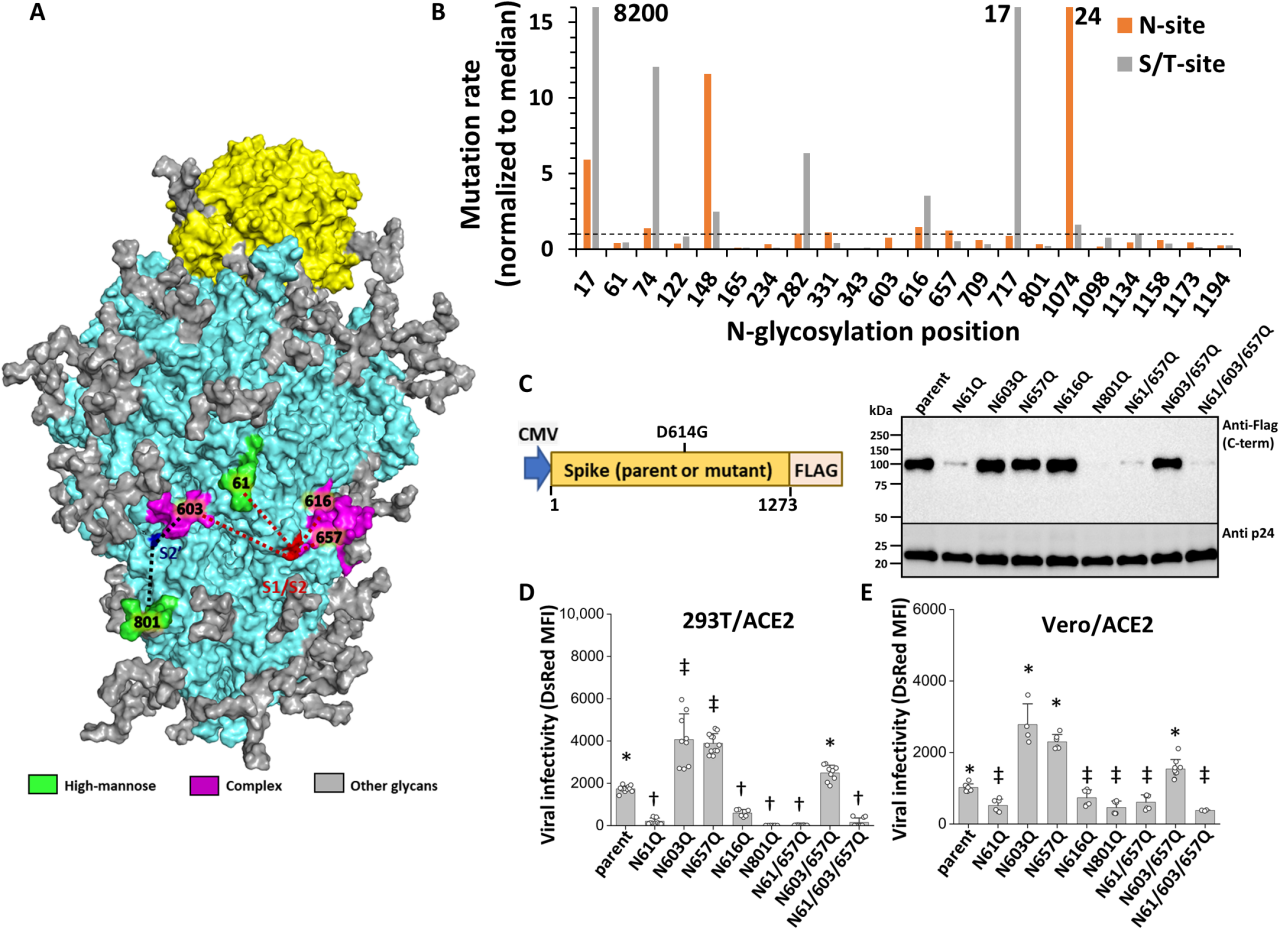
*N*-glycans on Spike are essential for pseudoviral entry. (**A**) Molecular model of Spike (cyan) bound to ACE2 (yellow). *N*-glycans are space-filled. Glycans studied in this work are color-coded either green (high-mannose) or magenta (complex structures) on the basis of published abundance data [Watanabe *et al.* (*5*)]. Distance of these glycans to S1-S2 (red) and S2′ (blue) cleavage sites are indicated using red and blue dashed lines. (**B**) Spike mutation rates at *N*-glycosylation sites (N-X-S/T) based on analysis of ~7.6 × 10^6^ genomic sequences from gisaid.org. Mutation rates are shown both at Asn (N) and Ser/Thr (S/T) sites in the *N*-glycosylation sequon. Median mutation rate across all amino acids is set to 1 (dashed line). (**C**) Pseudotyped lentivirus (PVs) were generated using the parent Spike (D614G mutant), along with a panel of *N*-glycosylation site single, double, and triple N-to-Q mutations on the same background. Western blot quantified Spike incorporation into virions on the basis of detection of C-terminal FLAG-tag present in all constructs. p24 capsid protein serves as loading control. (**D** and **E**) Viral infectivity was evaluated at 72 hours, using ACE2-overexpressing 293T [293T/ACE2, (D)] and Vero E6 cells [Vero/ACE2, (E)]. Mean fluorescence intensity (MFI) due to DsRed reporter is quantified. Data from multiple viral preparations were normalized to parent virus DsRed signal for the same batch. Data are means ± SD for *n* ≥ 3. *P < 0.05 with respect to all other treatments, †*P* < 0.05 with respect to parent, and ‡*P* < 0.05 with respect to all other treatments except that bars marked by ‡ are not different.

Our analyses focused on five *N*-linked glycans that are located proximal to either the S1-S2 proteolysis site (N61, N603, N657, and N616) or S2′ cleavage site (N603 and N801) of the Spike protein (Fig. 1A and table S1). These sites are almost fully occupied by a range of high-mannose and complex carbohydrate structures, when Spike protein is expressed in recombinant form, although the reported structures vary somewhat among the publications (*5*, *6*, *12*), as well as with the authentic virus (*24*). To study the functional role of these glycans, site-specific Asn (N) to Gln (Q) mutations were incorporated at each of these sites in the “parent” Spike protein containing the D614G mutation, i.e., NCBI: YP_009724390.1 with D614G. Constructs containing single (N61Q, N603Q, N657Q, N616Q, and N801Q), double (N61/657Q and N603/657Q), or triple (N61/603/657Q) mutations were generated. On the basis of analysis of 7.6 × 10^6^ Spike protein sequences downloaded from GISAID on 28 June 2022 (*25*), the natural mutation rates of all five residues is low, at or below the median mutation rate across the full Spike protein (Fig. 1B). This was particularly true for N61 and N801, where both the Asn residue and Ser/Thr located two bases away in the *N*-glycosylation sequon had very low mutation rates. In general, Spike *N*-glycosylation mutations do not appear to be tolerated by SARS-CoV-2, except for a few sites such as N17 and, possibly, N717 and N1074. Notably, the Omicron variant, despite its extensive number of nonsynonymous mutations across Spike, also does not carry mutations at *N*-glycosylation sites. Thus, several of the *N*-glycans may be necessary for viral fitness.

Lentivirus-based pseudovirus was generated using parent Spike and the eight Spike mutant constructs. Immunoblotting of viral lysates for the C-terminal FLAG-tag showed that the Spike protein in these virions was almost completely cleaved at the furin/“RRAR” polybasic site located at the S1-S2 junction, suggesting that S1 is often noncovalently associated with S2 in mature Spike (Fig. 1C) (*26*). Mutations at N61 and N801 reduced Spike incorporation into the pseudovirus, although some residual Spike could be seen upon overloading virus in lanes and developing for longer times (fig. S1A). Nevertheless, a reduction in Spike expression in N61Q and N801Q mutants was observed in all single, double, and triple mutants. This defect in Spike incorporation resulted in reduced pseudoviral entry in two-cell systems that overexpress human ACE2, 293T/ACE2 (Fig. 1D), and Vero/ACE2 (Fig. 1E). Among the remaining mutants, N616Q partially reduced viral entry, while N603Q and N657Q increased infectivity for reasons that are yet unknown. Percent DsRed-positive cell data for the same study are presented in fig. S1 (B to E), and these are consistent with Fig. 1 (D and E). Overall, specific *N*-glycans may regulate Spike maturation and viral entry function.

### SARS-CoV-2 VLP phenotype resembles the pseudovirus

**Fig. 2.**
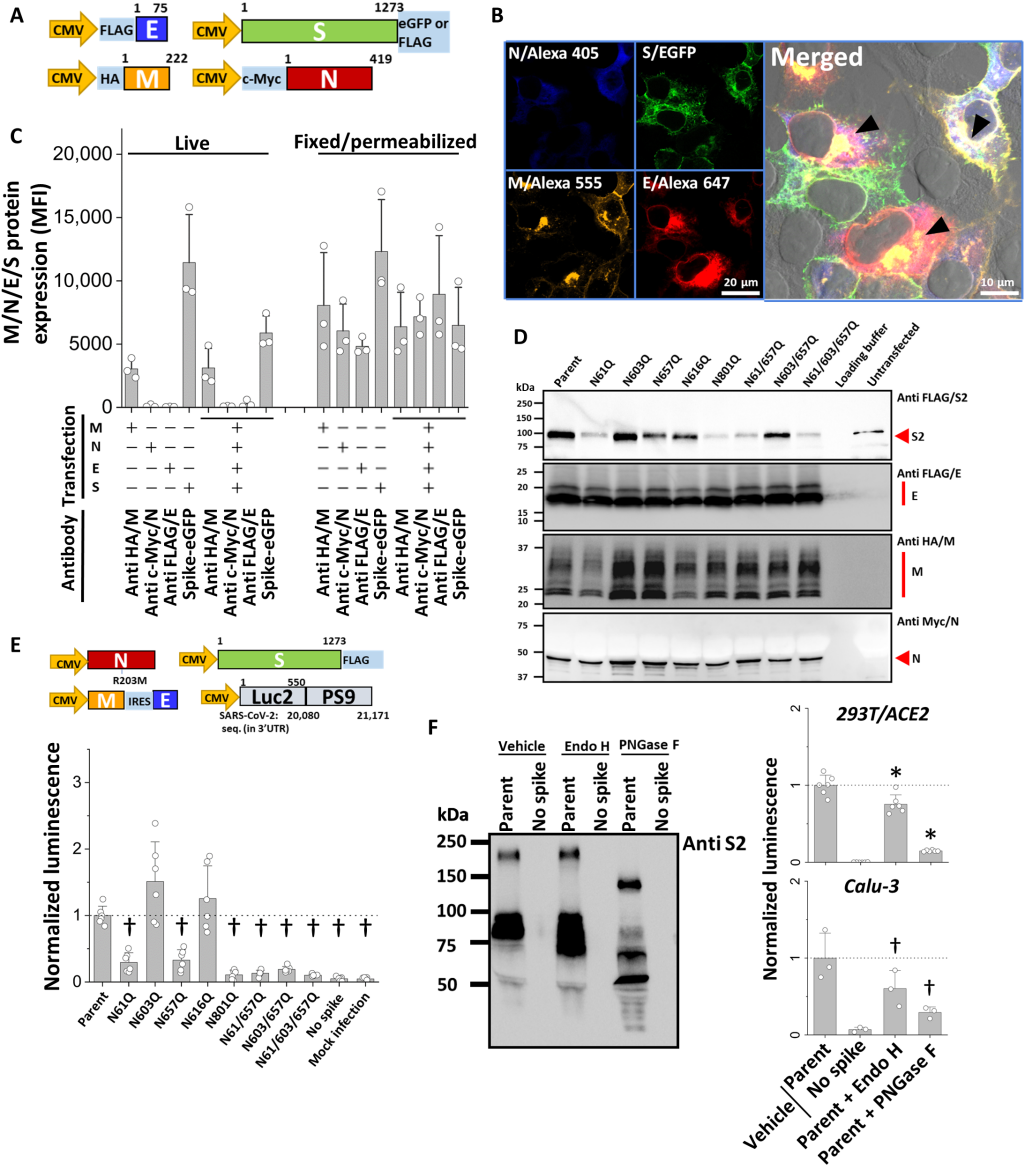
SARS-CoV-2 VLPs with *N*-glycan mutations display reduced viral entry. (**A**) Plasmids encoding for the four structural proteins containing distinct tags or EGFP reporters were either transfected alone or pooled and transfected together into 293Ts. (**B**) Confocal micrographs show the intracellular colocalization of M, N, E, and Spike in perinuclear compartments that likely includes the ERGIC (arrowhead). (**C**) Flow cytometry analysis performed with live and “fixed-permeabilized cells” demonstrate cell surface expression of Spike and M proteins. N and E were intracellular. Here, the structural proteins were either expressed alone or altogether: “+” and “−” indicate presence/absence of indicated proteins. (**D**) All proteins were expressed in VLPs (detected using epitope-tag Abs), but Spike carrying N61Q and N801Q mutations displayed somewhat reduced expression. (**E**) Luc-VLPs containing firefly luciferase reporter were produced by cotransfecting 293T cells with plasmids encoding for N (R203M mutant), M-IRES-E, Luc-PS9, and Spike constructs. Luc-VLP viral entry into 293T/ACE2 cells was reduced upon implementing many of the N-to-Q glycosylation mutations. “No-spike” VLPs were made using all plasmids except Spike. Mock infection did not contain VLP. Data were normalized to parent-VLP luminescence in each run [both for (E) and (F)]. (**F**) Luc-VLP with parent-Spike, produced using 293T cells, was deglycosylated using either Endo H or peptide *N*-glycosidase (PNGase) F. PNGase F reduced viral entry into 293T/ACE2 and Calu-3 cells, with a smaller effect being observed for Endo H. Data are means ± SD for *n* ≥ 3. *P < 0.05 with respect to all other treatments and †*P* < 0.05 with respect to parent.

We determined the degree to which observations made using pseudovirus that assemble at the cell membrane could be observed using SARS-CoV-2 VLPs that assemble in the ERGIC (endoplasmic reticulum Golgi intermediate compartment). To this end, VLPs were produced by transient cotransfection of 293T cells with four plasmids encoding for the SARS-CoV-2 structural proteins: envelope (E), membrane (M), nucleocapsid (N), and each of the panel of Spike variants. Many of these molecules were modified at the N terminus with molecular tags: FLAG-tag for E-protein, hemagglutinin (HA)–tag for M, and Myc-tag for N protein (Fig. 2A). All mutant Spike contained C-terminal FLAG as described in Fig. 1C, and an additional wild-type (WT) Spike–enhanced green fluorescent protein (EGFP) fusion protein was also used. Upon cotransfecting M, N, E, and Spike-EGFP plasmids into 293T cells, all proteins were detected in fixed cells (Fig. 2B and movie S1). Spike was observed to localize in distinct compartments that are presumably the Golgi, as well as the plasma membrane. The M-protein followed a similar pattern, although sites of more intense M staining were often observed in the perinuclear region. E was expressed closer to the nucleus, and the signal from N was diffused presumably because of its expression in the cytoplasm. Some colocalization of all constructs was observed in the perinuclear compartments, which likely includes the ERGIC. Single-protein transfection controls are presented in fig. S2A. Consistent with microscopy data, flow cytometry results suggest that M and Spike are expressed on the cell surface, as they could be readily detected in nonpermeabilized “live cells” (Fig. 2C). In contrast, N and E were exclusively intracellular and could only be detected following fixation and permeabilization. VLPs produced in serum-free media upon cotransfection with all four structural proteins were purified using ultracentrifugation. The particles sized at 120 to 200 nm using NanoSight tracking analysis (NTA) and ~100 nm based on negative-stain transmission electron micrographs (TEMs; fig. S2B). These particles were not detected in mock transfection NTA/TEM controls.

Western blots assessed the incorporation of parent Spike and the panel of Spike glycan mutants in the VLPs (Fig. 2D). Here, we observed E (8.5 kDa) and N (45.2 kDa) at approximately their expected molecular mass after accounting for the mass of their reporter-tags. M was detected as a set of bands in the range of 25 to 35 kDa, with mass variability likely due to N-terminal ectodomain glycosylation that has previously been reported in other coronaviruses (*27*, *28*). While the parent Spike was incorporated into VLPs, Spike incorporation was reduced in several of the *N*-glycan mutants including N61Q, N801Q, N61Q/N657Q, and the N61/603/657 triple mutant. The pattern was similar to that observed in the pseudovirus studies, although Spike bands were more clearly observed in the VLP studies in all constructs. VLP entry assays were performed by spinoculation as described previously (*29*), washing cells extensively; fix-permeabilizing them; and then measuring the presence of intracellular M, E, and Spike proteins (fig. S2, C and D). Viral protein quantitation was performed using flow cytometry, as the microscopy strategy suggested previously was not quantitative. Here, we observed that a fraction of the cells presented signal because of the viral proteins, only upon addition of the VLP and not the control that lacked the particles. The higher signal observed in the parent Spike VLP was reduced upon implementing the N801 mutation, and N61Q double and triple mutations (fig. S2C). While these data were mostly consistent with the pseudovirus data in Fig. 1, the measured signal was weak and it was difficult to discern between virus bound to the cell surface versus those that entered cells (fig. S2D).

To overcome the above limitations, we adopted the system recently described by Syed *et al.* (*30*), as it allows the incorporation of the firefly luciferase reporter in the VLP (“Luc-VLP”; Fig. 2E). These VLPs sized at ~100 to 120 nm on the basis of dynamic light scattering. Here, the entry of Luc-VLP–bearing parent-Spike into host 293T/ACE2 cells resulted in bright signal. VLP lacking Spike displayed low signal comparable to “mock infection” without any virus. In addition, implementing N61Q and N801Q mutations in Spike reduced Luc-VLP entry into 293T/ACE2, with additional inhibitory function also being observed upon implementing N657Q Spike mutation.

Besides the effect of glycans in regulating Spike maturation in virions, we determined whether glycans present on functional VLPs also contribute to viral entry (Fig. 2F). To this end, Luc-VLP–bearing parent-Spike was treated with peptide *N*-glycosidase (PNGase) F to release all *N*-glycans and endoglycosidase Endo H to remove only the oligomannose structures. Deglycosylation was complete in the case of PNGase F based on the observed molecular mass shift in Western blots, and it was partial in the case of Endo H, as high-mannose glycans only dominate a few sites, including N61 and N801 (Fig. 1A) (*5*, *8*). In functional studies, PNGase F treatment reduced SARS-CoV-2 VLP entry by 70 to 85% in 293T/ACE2 and Calu-3 cells. consistent with previous reports (*18*, *31*), while Endo H had a smaller impact on viral entry. These observations suggest that besides regulating Spike maturation and assembly into virions, both high-mannose structures and complex-type glycans may also directly contribute to Spike function during SARS-CoV-2 entry.

### Differential roles for *N*-glycans in virion packaging versus cell surface expression

**Fig. 3.**
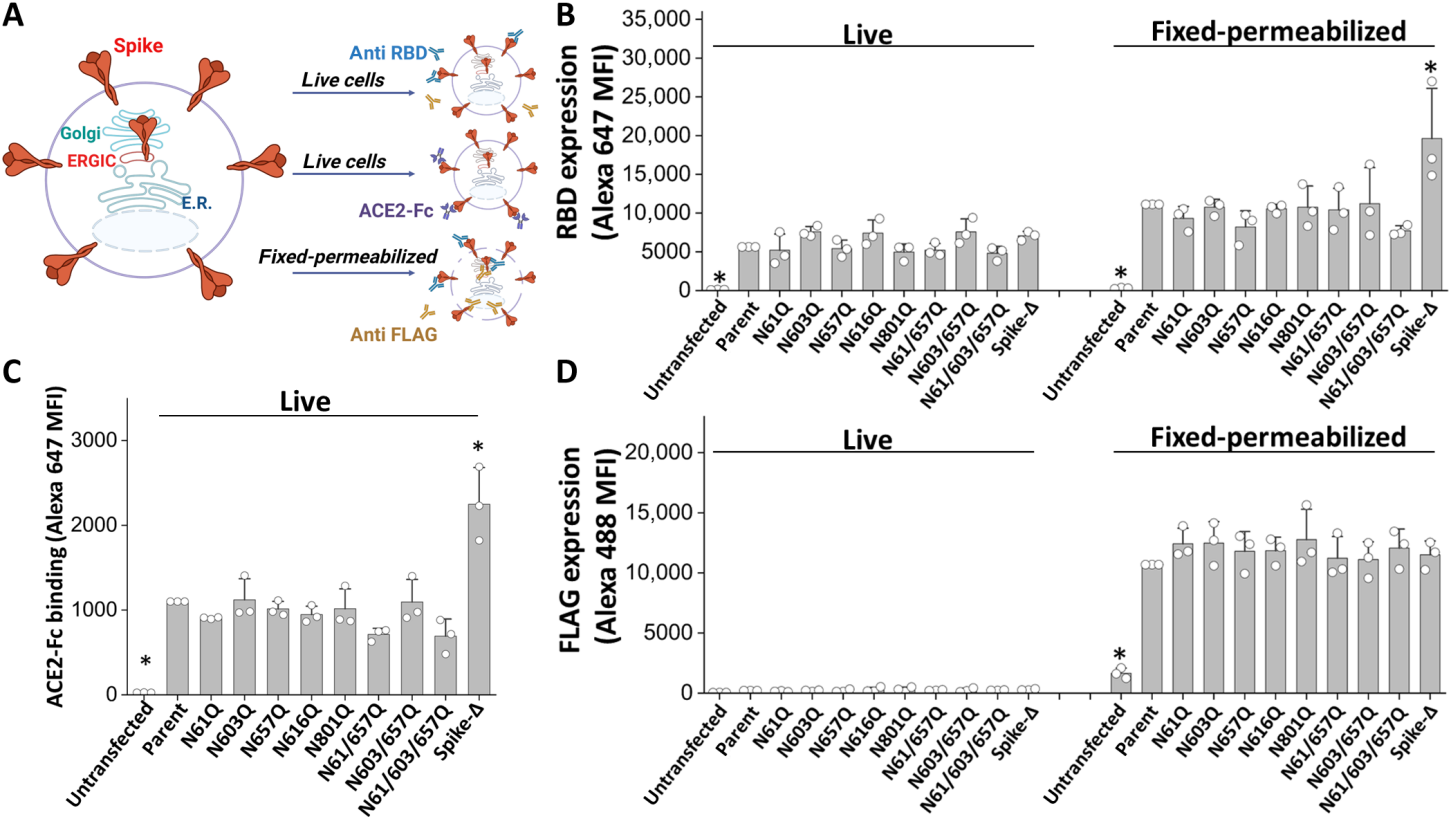
Glycan mutations did not affect Spike cell surface expression and ACE2 binding function. (**A**) Spike parent and mutants were transiently expressed in 293T cells. Spike cell surface expression was quantified on a portion of the live cells at 48 hours by measuring anti-RBD binding using flow cytometry. ACE2-Fc (0.7 μg/ml) binding was also independently quantified in live cells. A portion of the cells were “fixed-permeabilized” using paraformaldehyde-methanol method. Anti-RBD and anti-FLAG Ab binding quantified the presence of the Spike S1 and S2 subunits, respectively. (**B**) Spike S1 expression on 293T cells quantified using anti-RBD in live and fixed-permeabilized cells. (**C**) ACE2-Fc binding data in live cells. (**D**) Spike/S2 expression quantified using anti-FLAG Ab. All Spike variants were equally expressed on cell surface with Spike-Δ (without RRAR site) displaying more RBD/S1 presentation. ACE2-Fc binding correlated with RBD expression. Data are means ± SD (*n* = 3), after normalization with respect to parent Spike MFI in different runs. *P < 0.05 with respect to all other treatments.

We determined whether the above *N*-glycan site-specific mutations simply affected protein folding, which would then result in altered cell surface Spike expression and/or ACE2 binding function (Fig. 3). Thus, we used “live” cells to measure Spike S1 noncovalent association with S2 using an anti-RBD antibody (Ab) and quantified soluble ACE2-Fc fusion protein binding (Fig. 3A). In addition, we used fixed-permeabilized cells to quantify total Spike expression by measuring the level of the Spike intracellular C-terminal FLAG-tag. Here, we observed similar levels of cell-associated S1 for all *N*-glycan mutants, suggesting that these carbohydrates do not affect noncovalent S1-S2 association (Fig. 3B). Greater anti-RBD binding was detected in fixed-permeabilized cells, as this quantifies epitopes both inside and outside cells. The cell surface Spike proteins were similarly functional in all the glycosylation mutants, as they robustly bound soluble ACE2-Fc (Fig. 3C). Total Spike expression measured using anti-FLAG monoclonal Ab (mAb) was comparable in all cases (Fig. 3D). In the above study, ACE2-Fc binding to Spike-Δ was higher than other proteins since it lacks the polybasic furin/RRAR cleavage site (fig. S3). This results in higher S1/RBD subunit retention in Spike-Δ cells since S2 is expressed similarly in all cases. Overall, *N*-glycan site mutations at these five residues did not affect Spike expression on cells, S1-S2 association, or ACE2-Fc binding. This is different from the impact of N61 and N801 on Spike viral incorporation. Thus, the reduced viral entry noted previously upon implementing site-specific mutations is not due to the global reduction in Spike expression or RBD binding function.

### *N*-glycan mutations had a minor role on syncytia formation

**Fig. 4.**
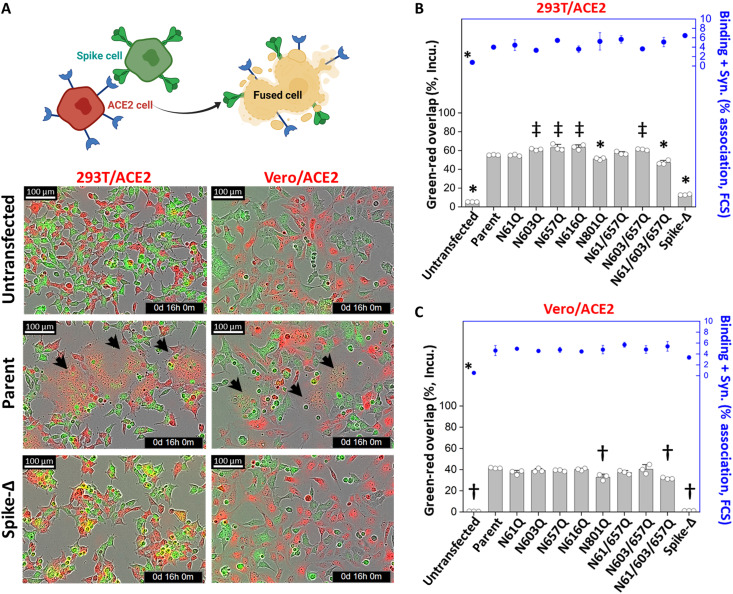
Small impact of Spike *N*-glycan mutations on syncytia formation. 293T cells were transiently transfected with the panel of Spike constructs to produce “293T/S.” Twenty-four hours after transfection, these cells were labeled using CMFDA (a green fluorescence dye) and mixed with either 293T/ACE2 or Vero E6 transfected to overexpress ACE2 (“Vero/ACE2”). These ACE2 cells were labeled with red fluorescence dye, CMTMR. Incucyte live cell imaging was performed during a 16-hour coculture experiment to monitor red-green dye overlap/fusion between Spike (green) and ACE2 (red)–expressing cells. A portion of the cells were also trypsinized at 4 hours for cytometry analysis. (**A**) Representative images for ACE2-expressing cells cocultured with either untransfected cells (top row) or cells expressing parent Spike (middle) or Spike-Δ proteins (bottom). Sixteen-hour images are presented for studies with 293T/ACE2 and Vero/ACE2. Arrow indicates syncytium. (**B** and **C**) The left axis presents percent green-red overlap [=100 × (overlap area/total area occupied by cells)], quantified using the Incucyte system at 16 hours. These data are presented for studies conducted with 293T/ACE2 (B) and Vero/ACE2 (C). Syncytia was observed in all cases, except for untransfected cells and cells expressing Spike-Δ. The right axis presents cytometry measured percent green-red events, i.e., % association = (green-red count/total count) × 100. This includes both cells that are bound to each other and those forming syncytium. Data are means ± SD for *n* = 3. *P < 0.05 with respect to all other treatments, †*P* < 0.05 with respect to parent, and ‡*P* < 0.05 with respect to all other treatments except that bars marked by ‡ are not different from each other.

Cleavage at the S2′ site triggers the exposure of the Spike fusogenic peptide that facilitates viral-cell membrane fusion and viral RNA release into host. As some of our glycans are located proximal to S2′, we determined whether these carbohydrates may affect cell-cell fusion using a syncytia formation assay adopted from the work of Papa *et al.* (*21*). To test this, WT 293T cells were transfected with the panel of Spike proteins for 24 hours before labeling with a green fluorescence dye CMFDA (5-chloromethylfluorescein diacetate). These cells were then mixed with 293T/ACE2 cells labeled using the red fluorescent dye CMTMR [5- (and-6)- (((4-chloromethyl)benzoyl)amino)tetramethylrhodamine]. Similar studies were also performed by mixing 293T-Spike cells with Vero E6 cells transiently transfected to overexpress ACE2 (Vero/ACE2). In both cell systems, we observed low or negligible syncytia formation when ACE2-bearing cells were mixed with cells lacking Spike or those expressing Spike-Δ (Fig. 4A and movies S2 and S3). This is consistent with the need for cleaving S1-S2 before S2′ proteolysis that promotes cell fusion. Expression of other Spike *N*-glycan mutants in both cell systems resulted in robust syncytia formation (Fig. 4, B and C, and movie S4). The only mutations that partially reduced syncytia formation were N801Q and N61/603/657Q, which displayed 10 to 20% reduction. The kinetics of syncytia formation was also similar in parent versus glycan-mutant Spike variants, as cell fusion could be detected in all cases at the earliest sampling time point (2 hours; fig. S4A). The data from live cell imaging were corroborated by flow cytometry analysis, performed at the 4-hour time point (Fig. 4, B and C). This assay enumerated the fraction of cell aggregate events displaying both red and green fluorescence signal, as this quantifies both Spike-ACE2 heterotypic cell binding and fusion (fig. S4B). Here, in addition, Spike mutations did not markedly affect the cytometry heterotypic cell aggregate measurements. Overall, among the *N*-glycan mutations, only the N801Q and N61/603/657Q mutants had a minor impact on S2′ cleavage and cell-cell fusion rates.

### Calnexin (*CANX*)/calreticulin (*CALR*) chaperones regulate Spike maturation and viral entry

The precise mechanism by which the Spike *N*-glycans reduce viral entry, yet retain function on cell surface, remains to be determined. As glycan-dependent partial reduction in Spike incorporation into virions was noted, we hypothesized that mammalian intracellular lectins may contribute to this observation (*32*). Furthermore, as *N*-glycosylation inhibitors targeting glucosidases and STT3A (catalytic unit of the oligosaccharyltransferase complex) have been shown to reduce SARS-CoV-2 infectivity (*17*, *18*, *31*), we determined whether ER-localized lectins related to these enzymes may contribute to Spike dysfunction in pseudovirus and VLP systems. To test this, CRISPR-Cas9 genome editing was used to generate isogenic 293T KO clones that lack the human lectins, calnexin (*CANX*) and calreticulin (*CALR*). These proteins aid the folding and quality control of glycoproteins in the ER by binding monoglucosylated glycoproteins. Nonhomologous end joining and frameshift mutations in these single-cell clones was confirmed using next-generation sequencing (fig. S5A). Flow cytometry performed using the fixation-permeabilization protocol confirmed that CANX and CALR expression was abolished in the respective KOs (fig. S5B). This was also further confirmed using Western blotting and specific mAbs that recognize these lectin chaperones (fig. S5C). Both KOs looked morphologically similar to WT cells and grew at similar rates, suggesting that a vast majority of growth and structure-associated proteins were intact. In addition, there was little change in the distribution of cell surface *N*-linked glycans as measured using a panel of lectins (fig. S5D): ConA (binds high mannose glycans); PHA-E/L (binds complex glycans); SNA [binds α(2-6)sialic acid]; AAL (binds terminal fucose); and RCA-1, ECL, and DSL (bind various aspects of lactosamine chains).

**Fig. 5.**
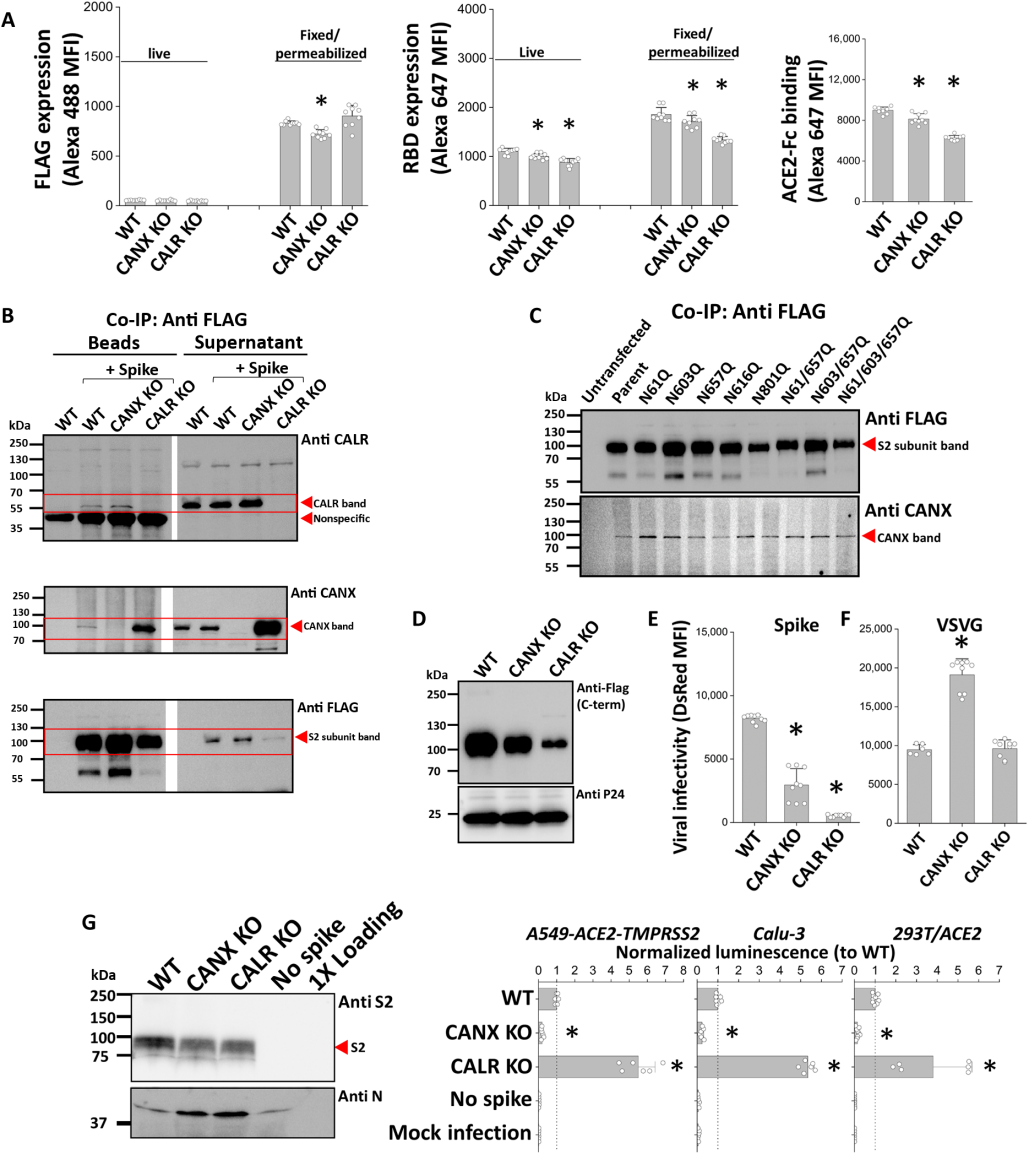
Chaperone proteins calnexin (*CANX*) and calreticulin (*CALR*) regulate SARS-CoV-2 viral entry. (**A**) Parent Spike was transfected into WT, *CANX*-KO, and *CALR*-KO 293Ts. Spike expression was similar in all cells based on anti-FLAG that detects the Spike C terminus (left). A partial (10 to 25%) reduction in anti-RBD Ab/Spike S1 association (middle) was noted, and this correlated with ACE2-Fc binding (right). (**B**) 293Ts were transfected with parent Spike. Spike immunoprecipitated from cell lysate 24 hours after transfection using anti-FLAG mAb and coprecipitated both CANX and CALR. CALR (top), CANX (middle), and Spike (bottom) were analyzed both in the immunoprecipitated (protein A/G “bead”) sample and the supernatant remaining after bead removal. Arrows and boxes indicate target proteins. (**C**) All Spike mutants coprecipitated CANX in the anti-FLAG Spike pulldown assay. Cells without Spike (“untransfected”) served as negative control. (**D**) Parent pseudotyped lentivirus was produced in WT, *CANX-*KO, and *CALR*-KO. Western blot showed reduced Spike incorporation into virions produced in both KOs. (**E**) Pseudovirus entry into 293T/ACE2, measured 72 hours after infection, was reduced when virus were produced in *CANX* or *CALR*-KO 293Ts. (**F**) VSV-G pseudotyped lentivirus entry into 293T/ACE2 was not reduced by either *CANX* or *CALR*. (**G**) Luc-VLP containing parent Spike were produced in WT, *CANX*-KO, and *CALR*-KO 293Ts. Luc-VLPs produced in *CANX*-KO displayed reduced entry into A549-ACE2-TMPRSS2, Calu-3, and 293T/ACE2 cells. Luc-VLP viral entry is reported on the basis of luminescence signal, normalized to parent levels in each run. No spike Luc-VLP lacks Spike. Data are means ± SD (*n* ≥ 3). *P < 0.05 with respect to all other treatments.

Upon expressing parent Spike in 293T, we observed comparable cell surface expression of the protein in WT cells and the two KOs, as determined using the anti-FLAG mAb that binds Spike C terminus (Fig. 5A). A small 10 to 25% reduction in RBD epitope or S1-subunit was observed in the KO cells, and this tightly correlated with similar reduction in ACE2-Fc binding. Thus, glycan modifications to Spike upon expression in *CANX*/*CALR* KOs may affect the stability of S1 noncovalent association with the S2 subunit. However, the *CANX*/*CALR* KOs do not appear to affect cell surface Spike expression or ACE binding. To determine whether Spike interacts with CANX and CALR within cells, coimmunoprecipitation (co-IP) experiments were performed in which Spike was immunoprecipitated using anti-FLAG Abs before Western blots were carried out to detect CANX/CALR association in these pull-downs (Fig. 5B). Here, the co-IP of Spike with both CANX and CALR were detected, confirming the physical association of these macromolecule pairs. In studies that determined whether any of the site-specific glycan mutations disrupt binding to the lectin chaperones, we observed similar association of CANX with the entire panel of parent and mutant Spike proteins (Fig. 5C). Similar studies could not be performed for CALR because of the proximity of this protein molecular mass with the mAb heavy-chain band, both at ~50 kDa.

To determine the role of these chaperones on viral entry, parent Spike pseudovirus generated in WT cells were compared with the same virion produced using the *CANX* and *CALR* KOs (Fig. 5, D and E). Here, Western blots showed reduced incorporation of Spike into virus produced in both *CANX* and *CALR* KO cells, with *CALR*-KO–derived virions displaying more severe reduction in Spike incorporation (Fig. 5D). Consistent with this, Spike pseudovirus produced from *CANX*-KO cells showed a 60% reduction in infectivity, while those produced using the *CALR-*KOs displayed an 80 to 90% reduction (Fig. 5E and fig. S6A). Changing the pseudovirus envelope protein to VSV-G (vesicular stomatitis virus G protein) (*9*) resulted in fully functional virus regardless of the producer cell type, WT 293T or *CANX*/*CALR*-KO (Fig. 5F and fig. S6, B and C). The expression of this glycoprotein receptor on virions enables broad host tropism, as it binds low-density lipoprotein receptors and phosphatidylserine that are ubiquitously present on many cells. Here, some enhancement in DsRed signal was observed when VSV-G pseudovirus was produced in *CANX*-KO cells (Fig. 5F), although the percent of DsRed-positive cells was unchanged (fig. S6C). These data suggest that the effect of CANX/CALR is specific to Spike incorporation into the pseudovirus rather than a global defect due to implementation of the CRISPR-Cas9 workflow.

Next, we compared Luc-VLPs produced in WT cells with those produced in *CALR*- and *CANX*-KOs (Fig. 5G). Here, in contrast to the pseudovirus, we observed minimal changes in Spike incorporation in Luc-VLPs regardless of the cell type used to produce them. Whereas Spike levels were similar in Luc-VLPs produced using *CALR*- and *CANX*-KOs, only the Luc-VLPs from the *CANX*-KO displayed ~80% reduction in viral entry into three different cell systems: 293T/ACE2, A549-ACE2-TMPRSS2, and Calu-3. Some enhancement in viral entry was consistently observed upon using Luc-VLPs from *CALR*-KO, and the mechanism for this remains under study. Overall, these data strongly support a major role for lectin chaperones in regulating viral entry for both the Spike-pseudotyped viral particles and SARS-CoV-2 Luc-VLPs. However, differences were also noted between the two model systems likely because the lentivirus buds from the cell membrane while the VLPs emerge from ERGIC.

## DISCUSSION

This study uses Spike-pseudotyped virions and SARS-CoV-2 VLPs to demonstrate a critical role for Spike protein *N*-glycans and the lectin chaperone calnexin in regulating viral entry. Among the glycan mutations, N61Q and N801Q displayed the most profound reduction in viral entry using both the pseudovirus and VLP systems, with a smaller effect of other residues. Thus, specific *N*-glycans on Spike appear to have distinct functional roles. This was observed using both 293T/ACE2 and Vero/ACE2 cells, two cell systems that not only support a cathepsin-based endocytic pathway (*33*) but also appear to exhibit some TMPRSS2 dependence (*21*). Whereas glycan site modifications had a major effect on viral entry, their effect on cell surface Spike expression, ACE2 binding, and syncytia formation was relatively smaller. This suggests that targeting glycosylation pathways in a therapeutic setting of SARS-CoV-2 infection would reduce direct viral entry, with less impact on cell-to-cell transfer of viral genome by fusion and related viral escape from humoral immunity (*34*).

Previous work support the concept that glycans are important regulators of viral function. In this regard, Li *et al.* (*10*) generated a panel of glycan mutations in Spike and observed that many of these resulted in less infectious particles, although the mechanism(s) of action was not explained. This included the N331Q and N343Q mutations that reduce ACE2 binding function and thus diminish pseudovirus infection of ACE2-bearing cells (*35*, *36*). Lu *et al.* (*11*) additionally report that several myeloid C-type lectins bind glycans located on the Spike S1 subunit at either the N terminus or C terminus of the RBD. This includes ASGR1 and CLEC10A, whose binding to Spike is reduced upon deleting a range of Spike NTD glycans, and DC-SIGN, L-SIGN, and LSECtin that are reported to bind N603 on Spike. Besides receptor-ligand binding, it is also suggested that some glycan deletions, specifically N165, N149, and N709, may increase viral sensitivity to neutralizing Abs presumably by exposing glycan-free Ab binding epitopes (*10*). While most of the above studies used pseudotyped viral particles, the current manuscript extends this using authentic SARS-CoV-2 VLPs. It demonstrates a critical role for *N*-glycans at N61 and N801 in regulating Spike maturation and related infectivity. It suggests that in addition to acting as a direct binding partner for lectins or acting as an immunological shield, the glycans of Spike also regulate other aspects of viral infectivity.

Upon making point mutations, we observed that selected glycan mutations reduced Spike incorporation into pseudovirus and VLPs, with less effect on cell surface expression. These findings are reminiscent of the report by Zhang *et al.* (*37*). These authors observed that the D614G Spike mutation enhances Spike incorporation into Maloney murine leukemia pseudovirus and SARS-CoV-2 VLPs, without affecting overall Spike expression on cells. The enhanced maturation of virion Spike protein in this study enhanced infectivity. In these studies, besides a direct role for Spike glycans in viral assembly, these carbohydrate structures may also interact with other host proteins that regulate viral assembly. A total of 300+ high-confidence protein-protein interactions have been reported between SARS-CoV-2 viral proteins and host cells (*38*). Some of these proteins may be glycosylated, and their expression, composition, and/or spatial distribution may be regulated by CANX/CALR. In this regard, while the exact proteins that are affected by CANX/CALR are yet to be cataloged, other related enzymes such as UGGT (uridine diphosphate–glucose:glycoprotein glucosyltransferases) are reported to assist the folding of 71 of the possible 4350 *N*-linked glycoproteins present in cells (*39*). Thus, the effects of the chaperone proteins could be specific. As a result, the pathways used for Spike viral encapsulation may be different from that regulating cell surface expression.

Upon studying the roles of *CALR* versus *CANX* using Spike pseudotyped lentivirus, we observed that viral infectivity tightly correlated with the ability of these chaperones to regulate Spike incorporation into pseudovirus, with CALR having a greater role compared to CANX. In contrast, SARS-CoV-2 Luc-VLP entry into ACE2-expressing cells was more strongly dependent on CANX, with the extent of viral entry being greater than that could be explained by changes in Spike incorporation into virions. In this regard, the overall concept that CANX is critical for viral function is consistent with other studies performed using hepatitis B (HBV M protein) (*40*), rabies (heterotrimeric guanine nucleotide–binding protein) (*41*), herpes simplex type 1 (HSV-1 gB, gC, and gD) (*42*), human cytomegalovirus (gB) (*43*), and the original 2002 SARS virus (Spike) (*44*). In addition, both CANX and CALR are thought to contribute to human immunodeficiency/HIV type-1 (gp160) (*45*) and influenza because of their impact on both HA and neuraminidase glycoproteins (*46*, *47*). Although CANX/CALR are typically considered to be ER/Golgi specific, these different viruses bud from different cellular compartments including the cell membrane (influenza, rabies, and HIV-1), nuclear envelope (HSV-1), multivesicular bodies (HBV), and ERGIC (Coronavirus) (*48*). In this regard, the difference that we observe, in pseudovirus versus VLP, could be due to the location of virus budding as the pseudovirus emerges from the cell membrane while VLPs bud from the ERGIC. In addition, CANX is membrane bound, while CALR is a soluble chaperone.

Having established the role of chaperone proteins, it is necessary to determine whether these molecules directly affect Spike pre- or postfusion structure. This is particularly relevant since the Luc-VLPs produced in *CANX*-KO displayed reproducible reduced (~80%) viral entry, with minimal change in viral Spike incorporation. It is also important to determine whether additional carbohydrate-related host proteins also regulate SARS-CoV-2 assembly. These potential candidates include other intracellular chaperones/folding-assist proteins (VIP36/*LMAN2*, BiP/*HSPA5*, endoplasmin, and glucosyltransferases UGGT1 and UGGT2), intracellular lectins (malectin), glucosidases (MOGS and GANAB), mannosidases, transport-associated proteins (ERGIC-53/*LMAN1*, MCFD2, and VIP36-like protein/*LMAN2L*), and molecules involved in Spike ER-associated degradation (OS-9, ERLEC1, and EDEM1 to EDEM3) (*32*, *49*, *50*). While the location of each of these proteins within cells under resting conditions is well established, their distribution can change quite markedly upon application of ER stress as shown for calnexin (*51*) and calreticulin (*52*). Consistent with this notion, a recent study shows that GRP78/BiP can be expressed on the cell surface and form a complex with Spike and ACE2 (*53*). The depletion of this chaperone reduces SARS-CoV-2 viral entry and infection. Additional mechanistic studies are needed to investigate these aspects, and validation is also desirable in related KO systems using the replicating virus.

Overall, our work shows that Spike *N*-glycosylation patterns, intracellular lectins and related molecular chaperones have unique roles in SARS-CoV-2 viral assembly and function. These glycan-related pathways may be targeted to modify SARS-CoV-2 viral entry, combat infection, and fight COVID-19 disease.

## MATERIALS AND METHODS

### Biochemicals

Rabbit anti-Spike RBD polyclonal Ab (pAb; catalog no. 40592-T62) and rabbit anti-p24 pAb (catalog no. 11695-RP02, RRID: AB_2904185) were from Sino Biologicals (China). Rabbit anti-SARS-CoV-2 nucleocapsid protein pAb (catalog no. 33336), rabbit anti-calnexin mAb C5C9 [immunoglobulin G (IgG); catalog no. 2679, RRID: AB_2228381], rabbit anti-calreticulin mAb D3E6 (IgG; catalog no. 12238, RRID: AB_2688013), and rabbit anti–β-actin mAb D6A8 (IgG; catalog no. 8457, RRID: AB_10950489) were from Cell Signaling Technology (Danvers, MA). Mouse anti-Myc tag mAb 2G8D5 (IgG2a; catalog no. A00704) and mouse anti-HA tag mAb 5E11D8 (IgG1; catalog no. A01244) were from GenScript (Piscataway, NJ). Rat anti-FLAG tag mAb L5 (IgG2a) in unconjugated (catalog no. 637301), Alexa Fluor 647–conjugated (catalog no. 637315, RRID:AB_2716154), and horseradish peroxidase (HRP)–conjugated (catalog no. 637311) forms were purchased from BioLegend (San Diego, CA). Mouse anti-SARS-CoV-2 Spike S2 subunit mAb 1034617 (IgG2A; catalog no. MAB10557-100), Alexa Fluor 488 mouse anti-HA tag mAb 912426 (IgG1; catalog no. IC6875G-100UG), and Alexa Fluor 647 rabbit anti-FLAG tag mAb 1042E (IgG; catalog no. IC8529R-100) were from R&D Systems (Minneapolis, MN). Secondary Abs used in Western blots and flow cytometry include HRP-conjugated goat anti-mouse IgG (catalog no. 115-035-068, RRID: AB_2338505), HRP-conjugated donkey anti-rabbit IgG (catalog no. 711-035-152, RRID: AB_10015282), Alexa Fluor 647 goat anti-human-Fc IgG (catalog no. 109-605-008, RRID: AB_2337882), and Alexa Fluor 647 mouse anti-rabbit IgG pAbs (catalog no. 211-605-109, RRID:AB_2339172) from Jackson ImmunoResearch (West Grove, PA). All lectins were purchased from Vector Laboratories (Burlingame, CA). If necessary, then Ab labeling was performed in phosphate-buffered saline (PBS; pH 7.4) by addition of 15-fold molar excess of succinimidyl ester coupled Alexa Fluor 488, Alexa Fluor 555, or Alexa Fluor 647 dyes (Fluoroprobes, Scottsdale, AZ). Following incubation for 1 hour at room temperature (RT), the reaction was quenched with 1/10th volume 1 M tris-HCl, and unreacted Alexa Fluor dye was removed using 7-kDa molecular mass cutoff Zeba desalting spin columns (Thermo Fisher Scientific). All other biochemicals were from Thermo Fisher Scientific (Waltham, MA) or Sigma-Aldrich (St. Louis, MO), unless otherwise mentioned.

### Cell culture

Human embryonic kidney 293T Lenti-X cells (“293T”; catalog no. 632180) were purchased from Clontech/Takara Bio (Mountain View, CA). Stable 293T/ACE2 cells were provided by M. Farzan (Scripps Research, Jupiter, FL). African green monkey Vero E6 cells (catalog no. CRL-1586) and Calu-3 human airway epithelial cells (catalog no. HTB-55) were from the American Type Culture Collection (Manassas, VA). All cells were cultured using Dulbecco’s modified Eagle’s medium supplemented with 10% fetal bovine serum, 1% antibiotic-antimycotic, and 1% GlutaMAX supplement. Cultures of Calu-3 were additionally supplemented with 1% non-essential amino acids, and 293T/ACE2 cultures contained puromycin (1 μg/ml). A549 lung carcinoma overexpressing human ACE2 and TMPRSS2 (A549-ACE2-TMPRSS2; catalog no. a549-hace2tpsa) were purchased from InvivoGen (San Diego, CA) and cultured according to the manufacturer’s instruction. All cell culture was performed in incubators maintained at 37°C, humidified, 5% CO_2_ environment. All cells were mycoplasma negative.

### Molecular model

Molecular dynamics simulations of ACE2-Spike complex were described previously (*9*). The distance between *N*-glycosylation sites and S1-S2 or S2′ proteolysis sites were estimated using intermediate structures from these simulations. The position of glycans in all three Spike chains in trimeric complex and their distances with all three S1-S2 and S2′ sites were considered when quantifying interatomic distance (table S1).

### Spike mutation frequency

Spike protein sequence data in .fasta format were provided by GISAID.org (*25*). Sequences with more than four base deletions and three unidentified nucleotides were removed from the dataset to focus on more complete sequence records. Remaining sequences were aligned against the parent Spike sequence using the Needleman-Wunsch global alignment algorithm (MATLAB code available at https://github.com/neel-lab/SpikeAnal). Mutations at each amino acid residue were counted. Median mutation rates across all amino acids were also calculated. Mutations that result in loss of *N*-glycosylation due to alteration of either N or S/T in the N-X-S/T glycosylation sequon were quantified.

### Molecular biology

The plasmid containing the original full-length SARS-CoV-2 Spike protein (“Spike-WT”), with C-terminal FLAG-tag, was provided by M. Farzan (*9*, *37*). The VSV-G envelope, and a vector coexpressing full-length human ACE2 with blue fluorescence protein reporter (Addgene 164219), was available from prior work (*9*). The site-directed D614G mutation was implemented on Spike-WT to yield the parent plasmid used in this work. Additional N-to-Q mutations were implemented on the parent to ablate specific *N*-glycosylation sites: N61Q, N603Q, N657Q, N616Q, N801Q, N61/657Q, N603/657Q, and N61/603/657Q. Four amino acids encoding for the S1-S2 polybasic cleavage site (RRAR) in the parent Spike protein were deleted in the Spike-Δ construct. Plasmids expressing SARS-CoV-2 structural proteins, with N-terminal tags, were provided by E. O. Sapphire (The La Jolla Institute for Immunology, La Jolla, CA), and these were used to make VLPs. These include M-protein with HA-tag (pcDNA3.1 + HA-SARS2-M), E-protein with FLAG-tag (pCMV+3x FLAG-SARS2-E), and N-protein with Myc-tag (pcDNA3.1 + Myc-SARS2-N). Plasmid expressing Spike-EGFP was obtained from GenScript (MolecularCloud, MC_0101089). The CoV2-M-IRES-E (Addgene plasmid no. 177938), CoV2-N-R203M (Addgene, 177952), and Luc-PS9 (Addgene, 177942) plasmids were gifts from J. Doudna (*30*). These plasmids were used to produce Luc-VLPs.

### Flow cytometry

Cells (10^7^/ml) suspended in PBS were incubated with either concentration of various Abs (0.5 to 5 μg/ml) or ACE2-Fc fusion protein (4 μg/ml) for 15 to 20 min on ice. Following, a brief wash using PBS, 1:200 to 1:500 diluted secondary fluorescent Abs were added if necessary for an additional 15 to 20 min. Samples were then again washed with PBS and read immediately using a BD LSRFortessa X-20 flow cytometer (San Diego, CA). In some cases, a fixation-permeabilization method described previously was adopted to characterize both extra- and intracellular protein epitope expression (*54*). Here, 0.5% paraformaldehyde (J.T. Baker) was added to 10^7^ cells/ml suspended in PBS for 1 hour at RT. The cells were then washed twice with PBS containing 1% bovine serum albumin (BSA) and resuspended in 100% anhydrous methanol for permeabilization at 4°C for 10 min. Following two additional wash cycles with PBS containing 1% BSA, Ab incubation was performed as described above.

### Transfection and protein expression

293T cells were transfected using the calcium phosphate precipitation method described previously (*55*). Plasmids were introduced into Vero E6 cells using Fugene-HD transfection reagent (Promega) according to the manufacturer’s instructions. These methods were used to express recombinant Spike and human ACE2 on cell surface, and to produce soluble recombinant ACE2-Fc protein. In both cases, cells were plated in either six-well plates or 150-mm tissue-culture grade petri dishes. Following overnight culture to reach ~70% confluence, fresh culture medium was added 1 hour before transfection. Whereas transfection of six-well plate required 3 μg plasmid per well, this was increased to ~45 μg per plate in the case of 150-mm petri dishes. In all cases, the transfection reagent was removed 6 to 8 hours after transfection by replenishing fresh serum–reduced Opti-MEM media (Thermo Fisher Scientific). In the case of protein production, the supernatant was centrifuged at 2000*g* × 2 min to remove cellular debris and then the protein was concentrated by 50-kDa cutoff centrifugal filters and stored at 4°C for downstream application.

### CRISPR-Cas9 KO cells

*Streptococcus pyogenes* Cas9–directed sgRNA (single-guide RNA) optimized for high on-target and low off-target genome editing of target genes (*CANX* and *CALR*) were obtained from integrated DNA Technologies (Coralville, IA). The sequences were “TCGTATAAGGGGTCTTGTCA” and “TATACTTCCCCTGTTGGAAC” for CANX and “CGGCAAGTTCTACGGTGACG” and “GCCGTCTACTTCAAGGAGCA” for CALR. These sgRNA were cloned into the px330 vector [Addgene 42230; (*56*)] as described previously (*57*). Both plasmids targeting a single gene were pooled and transfected into 293T cells using the calcium phosphate method. Single isogenic clones were obtained using fluorescence-activated cell sorting, without any sorting markers. Following scale up, cells were fixed using 0.5% paraformaldehyde and permeabilized with 100% anhydrous methanol. Intracellular protein flow cytometry was performed to screen for colonies using anti-CANX or anti-CALR Abs as described above. Western blots were also performed to confirm the KOs. For additional confirmation, genomic DNA were obtained from these clones using the PureLink Genomic DNA Isolation Kit (Thermo Fisher Scientific); the target regions were polymerase chain reaction–amplified (primers in table S2), barcoded with standard Nextera Index primers, and subjected to either 150–base pair (bp) or 300-bp paired-end sequencing on a MiSeq instrument (Illumina, San Diego, CA) (*58*).

### Pseudovirus production, titration, and viral entry assay

Pseudovirus was produced using the third-generation lentivirus system by replacing the standard VSV-G envelope protein with various Spike mutants (*59*). To produce pseudovirus, 293T cells (Takara Bio, MountainView, CA) were seeded in 150-mm petri dishes on day 0. Upon reaching ~70% confluence the following day, fresh medium was added 1 hour before transfection. The calcium phosphate precipitation method described above was used for transfecting with 49.2 μg of plasmids (18.7 μg of pLKO.1 TRC-DsRed, 21.9 μg of psPAX2, and 8.6 μg of various Spike vectors). Six to eight hours after transfection, the medium was changed to Opti-MEM for virus production. The first virus batch was collected 18 to 20 hours later. Fresh Opti-MEM medium containing 10 mM sodium butyrate (Sigma-Aldrich) was then added, and a second virus batch was collected 16 to 20 hours thereafter. These two batches were then pooled, centrifuged at 2000*g* × 2 min, and filtered through 0.45-μm polyethersulfone membrane to remove cell debris. The collected supernatant was then ultracentrifuged at 50,000*g* for 2 hours at 4°C to form the viral pellet. This final product was carefully resuspended in 100 μl Opti-MEM, aliquoted and stored at −80°C. The pseudoviral titers were determined using the lentiviral p24 Enzyme-linked Immunosorbent Assay Kit from Takara Bio (MountainView, CA) following the manufacturer’s instructions.

During the pseudovirus entry assay, 293T/ACE2 cells and transiently transfected Vero/ACE2 cells were trypsinized and resuspended in Opti-MEM medium. Pseudovirus were added to 30,000 293T/ACE2 or Vero/ACE2 cells suspended in Opti-MEM containing polybrene (8 μg/ml) in 1.5-ml Eppendorf tubes to reach a total 25-μl volume. The virus conc was p24 equiv. (0.6 μg/ml) for 293T/ACE2 and p24 equiv. (15 μg/ml) for Vero/ACE2. Following a 25-min incubation at RT, additional 200-μl culture medium was added, and the cells were plated in 48-well plates for an additional 72 hours. These cells were then trypsinized, spun down, resuspend in PBS buffer, and then directly analyzed using flow cytometry. Mean fluorescence intensity (“MFI”) of the entire cell population was recorded in the DsRed-channel. Percent DsRed-positive cells was also measured. If necessary, then human ACE2-expressing cells were subgated before the above analysis.

### VLP (fluorescence detection)

A total of 7 × 10^6^ 293T cells were plated in 100-mm petri dishes on day 0. The next day, the cells were cotransfected with M, N, E, and Spike plasmids at an equimolar concentration to total 24 μg of plasmid using the calcium phosphate method. Some of these plasmids were epitope-tagged. Media was switched to Opti-MEM 6 to 8 hours later for production of VLPs. The supernatant was then collected 60 hours after transfection and centrifuged at 2000*g* × 2 min to remove cell debris.

For the particle sizing studies, the clarified supernatant was carefully layered on top of an equal volume of 20% sucrose cushion. This was spun in an ultracentrifuge (Beckman Type 70 Ti rotor) at 100,000*g* for 3 hours, 4°C. VLP pellets thus formed were resuspended in TNE buffer (50 mM tris-HCl, 100 mM NaCl, and 0.5 mM EDTA, pH 7.4) containing 5% sucrose. The VLP sample was aliquoted and stored at −80°C if necessary. Electron microscopy and NanoSight analysis were performed with these samples. To size the VLPs, in some cases, VLPs prepared above were diluted at 1:100 in PBS, and 400 μl of this sample was injected into the sample chamber of a NanoSight LM10 instrument using a 1-ml syringe. Nanoparticle size was recorded using the NanoSight NTA 2.3 software: camera level, 11; screen gain, 7.00; and detection threshold, 10 pixels. In other cases, VLPs washed with water (2×) were negative-stained and imaged using a Hitachi HT7800 High Resolution 120-kV TEM.

The protocol for VLP entry followed from prior work (*29*). Briefly, 5 ml of clarified VLP containing culture supernatant described above were added to 293T/ACE2 cells in six-well plates. The VLPs were spinoculated into cells by centrifugation at 2000 rpm for 1 hour at 4°C. Following additional 2-hour culture in a standard tissue-culture incubator, these cells were washed with PBS, trypsinized, and resuspended in PBS. Paraformaldehyde (0.5%) was then added to fix the cells at RT for 1 hour. Following a wash with PBS containing 1% BSA, the cells were permeabilized using 100% methanol for 10 min at 4°C. Two more washes were performed using PBS containing 1% BSA before addition of fluorescent anti-FLAG Ab to detect Spike and E-protein, and fluorescent anti-HA to detect M protein for 10 min. These cells were then extensively washed and analyzed using flow cytometry.

### VLP-containing luciferase reporter (Luc-VLP)

The Luc-VLP platform was adopted from a recent publication (*30*), with minor modifications. To produce these particles, 18 × 10^6^ 293T cells were plated in 150-mm petri dishes on day 0. The following day, the cells were cotransfected with total mass of 50 μg of four plasmids at defined mass ratios: M-IRES-E:N:Luc-PS9:Spike::0.165:0.335:0.5:0.02. Six to eight hours after transfection, the culture medium was switched to Opti-MEM (Thermo Fisher Scientific). Forty-eight hours after transfection, the VLP containing supernatant was collected, spun at 2000*g* × 5 min to remove any cell debris and then filtered through 0.45-μm polyethersulfone membrane.

For the sizing studies, VLPs were purified from the clarified supernatant using the above sucrose-gradient ultracentrifugation procedure with minor modifications: The spin speed was 180,000*g* for 2 hours at 4°C, and the pellet was resuspended in 150 μl of PBS. Luc-VLP obtained in this manner were diluted 1:100 and sized using a DTS0012 sample cuvette placed in an Zetasizer Ultra instrument (Malvern Panalytical, Malvern, UK). Equilibration time was set to 120 s, temperature was 25°C, angle of detection was selected as back scatter, analysis model was general purpose, and all other settings were kept at default.

For VLP entry studies, the clarified cell culture supernatants (20 ml) were concentrated 100-fold using 100-kDa cutoff concentrator cartridges (Thermo Fisher Scientific). In some cases, the concentrated Luc-VLPs were deglycosylated using Endo H or PNGase F from New England Biolabs (Ipswitch, MA) following the manufacturer’s instruction. Here, 250 μl of concentrated Luc-VLP were incubated with 1:10 volume (i.e., 50,000 U/ml final) of either of the glycosidases for 6 hours at 37°C. The control VLP was treated identically with vehicle. For the entry assay, 50 μl of these above concentrated VLPs were mixed with 80,000 target cells (293T/ACE2, Calu-3, or A549-ACE2-TMPRSS2) at RT for 20 min in microcentrifuge tubes. The cells were then plated into 96-well tissue culture plates for overnight culture with 100 μl of additional medium. The next day, the supernatant was aspirated, the cells were gently washed once with warm PBS, and then lysed using 50 μl of mammalian cell lysis buffer (Gold Biotechnology, St. Louis, MO) for 20 min at RT with shaking. Fifty microliters of the produced cell lysate was then transferred into white round bottom 96-well plates, mixed with 50 μl of 2X TMCA luciferase assay buffer [10 mM MgCl_2_, 0.5 mM coenzyme A, 0.3 mM adenosine triphosphate, and 200 mM tris-HCl (pH 7.8)] along with 1 μl of 100X GLSS d-luciferin substrate. Luminescence intensity was measured immediately using a BioTek Synergy4 plate reader (Santa Clara, CA). All luminescence data in a single run were normalized with respect to VLP-bearing parent-Spike proteins.

### Western blot

Each virus sample was denatured in SDS-dithiothreitol loading buffer (Cell Signaling Technology) by heating at 98°C for 5 min. A total of 10^6^ cells were similarly lysed by mixing with 100 μl of SDS loading buffer, sonicating for 20 s, followed by denaturation by heating. Forty nanograms of p24 equivalent of pseudovirus, 10 μl of VLPs concentrated 100-fold using a 100 K cutoff centrifugal device (Thermo Fisher Scientific), and 5 μl of cell lysates were then resolved using a 12% tris-glycine gel. Five microliters of glycosidase-treated VLPs were resolved using a 4 to 12% tris-glycine gel. In the case of co-IP, ~10^6^ 293T cells transiently transfected to express Spike for 48 hours were washed and lysed using 500 μl of IP lysis buffer (Thermo-Pierce) containing 1 × Halt protease inhibitor (Thermo Fisher Scientific) for 5 min on ice with periodic mixing. The lysate was then spun down at 13,000*g* × 10 min, and ~500 μl of supernatant was collected and incubated with 15 μl of protein-A/G agarose beads (Millipore Sigma) for preclearing at RT for 1 hour. Simultaneously, in a separate tube, 10 μl of anti-FLAG mAb (0.5 mg/ml) was added to 15 μl of agarose protein-A/G beads, also for 1 hour. At 1 hour, precleared supernatant was spun down (13,000*g* × 5 min), collected from the first tube and added to the second tube. Following overnight incubation at 4°C with shaking, the bead-complex was spun down at 13,000*g* × 5 min. The supernatant was collected to measure unbound Spike, and the pelleted beads were washed extensively to release loosely bound components. Both the collected supernatant and beads were reduced and resolved using 12% tris-glycine gel. Following transfer onto nitrocellulose membrane, the membranes were blocked for 1 hour at RT using TBST solution (100 mM sodium chloride, 20 mM tris-HCl, and 0.1% Tween 20) containing 5% nonfat milk. This was followed by overnight 4°C incubation with primary Ab in TBST containing 2% milk. The next day, the membranes were washed with TBST solution (4 × 5 min) and, if necessary, then incubated with HRP-conjugated secondary Ab (1:1000 to 1:2000) for 1 hour at RT. Following extensive washing, signal was developed using the SuperSignal chemiluminescence substrate (Thermo Fisher Scientific) and images were acquired using a ChemiDoc Imaging System (Bio-Rad, Hercules, CA). Primary Abs were used in Western blots at 1:1000 to 1:2000 dilution of the original stock.

### Confocal microscopy

Four-well glass chamber slides (Thermo Fisher Scientific) were pretreated with Matrigel (10 μg/ml) diluted in PBS for 1 hour at 37°C. The slides were then rinsed with PBS once and seeded with 0.2 × 10^6^ 293T cells. The following day, the cells were transfected with 0.75 μg of M, N, E, and Spike-EGFP individually or cotransfected with all four plasmids in equal amounts (total, 0.75 μg). Medium was switched to Opti-MEM 6 to 8 hours later. Twenty-four hours after transfection, the cells were washed using PBS, fixed with 4% paraformaldehyde for 15 min at RT, rinsed 3× using PBS, and then permeabilized for 1 hour with Ab dilution buffer (PBS + 1%BSA + 0.3% Triton X-100). Following a wash, the cells were permeabilized-stained overnight by addition of Ab dilution buffer containing fluorescent anti-HA, anti-Myc, and anti-FLAG mAbs. The next day, the cells were extensively washed with PBS three times for 5 min each. ProLong diamond anti-fade reagent (Thermo Fisher Scientific) was then applied. The slides were then imaged using a Zeiss LSM 710 confocal microscope (40×/1.4 oil DIC objective, Ex:Em = 405:413 to 469, 488:501 to 534, 550:560 to 609, and 633:648 to 750).

### Syncytia formation

About 3.5 × 10^6^ 293T/ACE2 or ~1.3 × 10^6^ transiently transfected Vero/ACE2 in six-well plate (40 hours after transfection of human ACE2 protein) were labeled using 2 μM CellTracker Orange CMTMR (Thermo Fisher Scientific) diluted in Opti-MEM according to the manufacturer’s protocol. About 3.5 × 10^6^ 293T cells transiently expressing various Spike proteins were labeled using 2 μM CellTracker Green CMFDA (Thermo Fisher Scientific) diluted in Opti-MEM. A total of 10^5^ ACE2-expressing cells per well were then mixed with 10^5^ Spike-expressing cells per well in 24-well plates. The individual wells were imaged in a standard cell culture incubator over 16 hours, at 2-hour intervals, using the Incucyte S3 Live-Cell Analysis System (Sartorius, Germany). The acquired images were analyzed using Incucyte 2021A software to evaluate syncytia formation efficiency (syncytia percentage = 100% × green-red overlap area/total area occupied by cells). In some cases, the above experiment was stopped at 4 hours, the monolayer was washed using PBS and trypsinized, and then, the cells were analyzed using flow cytometry. Here, a gate was set for events that were larger than singlets. Percent association quantified the percent events in this subgate with mixed red and green fluorescence as they measured the number of tightly bound cells including syncytia events (% association = 100 × #red-green nonsinglet events/total number of events).

### Statistical analysis

All data are presented as means ± SD. All comparisons were performed using analysis of variance (ANOVA) followed by the Student-Newman-Keuls posttest. *P* < 0.05 was considered to be statistically significant. The number of repeats is specified in individual panels using discrete points. All presented data are biological replicates. While, typically, multiple virus or reagent batches were generated to ensure reproducibility, a single virus batch was used for up to three replicates in any given panel. Western blots are mostly representative of three or more biological replicates, particularly for the pseudovirus and VLP blots.

## Notes

View/request a protocol for this paper from *Bio-protocol*.
