# Supplementary material for: Role for N-glycans and calnexin-calreticulin chaperones in SARS-CoV-2 Spike maturation and viral infectivity
Source: Sci Adv. 2022 Sep 23;8(38):eabq8678. doi: 10.1126/sciadv.abq8678 (PMC9506717; doi:10.1126/sciadv.abq8678)
Supplement: Supplementary file 1 — Tables S1 and S2 Figs. S1 to S6 [file sciadv.abq8678_sm.pdf]

Science Advances
AAAS

Supplementary Materials for

Role for *N*-glycans and calnexin-calreticulin chaperones in SARS-CoV-2 Spike maturation and viral infectivity

Qi Yang *et al.*

Corresponding author: Sriram Neelamegham, neel@buffalo.edu

Sci. Adv. **8**, eabq8678 (2022)
DOI: 10.1126/sciadv.abq8678

The PDF file includes:

Tables S1 and S2
Figs. S1 to S6
Legends for movies S1 to S4

Other Supplementary Material for this manuscript includes the following:

Movies S1 to S4

SUPPLEMENTAL MOVIES

Movie S1. Confocal microscope stack of cells expressing E (Alexa-647, red), N (Alexa-405, blue), M (Alexa-555, orange) and Spike (EGFP, green) proteins.

Supplemental Movies S2-S4: Movies showing syncytia formation. At the starting point, 293T cells expressing Spike were labeled green while cells expressing ACE2 (293T or Vero E6) were labelled red. Cell fusion results in the appearance of multinucleated cells that are yellow colored. Each movie is 16h long with images captured at 2h intervals. All data were acquired using an Incucyte S3 fluorescence live-cell analysis system (Sartorius, Germany) at 10X magnification.

Movie S2. 293T/ACE2 with a) 293T Spike-parent cells, b) 293T Spike-Δ cells, and c) untransfected 293T cells.

Movie S3. Vero/ACE2 with a) 293T Spike-parent cells, b) 293T Spike-Δ cells, and c) untransfected 293T cells.

Movie S4. 293T/ACE2 with various Spike expressing cells: a) 293T Spike-N61Q, b) 293T Spike-N603Q, c) 293T Spike-N657Q, d) 293T Spike-N616Q, e) 293T Spike-N801Q, f) 293T Spike-N61Q/N657Q, g) 293T Spike- N603Q/N657Q, and h) 293T Spike-N61Q/N603Q/N657Q.

SUPPLEMENTAL TABLES

Table S1: Distance from N-X-S/T glycosylation site to cleavage sites (Å units) *

A. acid	Distance to S1/S2	Distance to S2'
61	32.72 ± 4.20	64.96 ± 1.07
74	46.98 ± 7.19	85.04 ± 2.67
122	60.92 ± 5.93	75.65 ± 1.51
149	76.63 ± 3.31	95.25 ± 10.26
165	73.70 ± 5.97	80.57 ± 2.15
234	58.73 ± 5.16	74.24 ± 2.45
262	46.61 ± 4.56	73.88 ± 1.21
323	43.88 ± 2.96	73.46 ± 0.86
325	51.63 ± 3.02	76.84 ± 0.44
331	67.24 ± 3.12	91.92 ± 0.25
343	86.59 ± 4.89	103.24 ± 5.30
603	23.74 ± 4.39	39.09 ± 1.67
616	33.08 ± 3.65	64.01 ± 1.57
657	19.02 ± 5.29	57.25 ± 0.97
709	50.92 ± 4.70	59.80 ± 0.55
717	42.27 ± 3.01	39.73 ± 0.22
801	48.56 ± 1.16	19.35 ± 0.88
1074	43.16 ± 4.24	52.18 ± 0.35
1098	52.51 ± 4.43	56.18 ± 0.45
1134	64.75 ± 4.28	66.11 ± 0.41

* linear distance between N-glycosylation site and S1-S2 or S2' cleavage site was calculated in the Spike trimer for all chains. Average distance (± STD) is presented. While N61, N603, N616 and N657 were proximal to S1-S2 (green highlight), N603 and N801 were proximal to S2' (green highlight).

Table S2: Primers used to generate NGS amplicons for sequencing *CANX-KO and CALR-KO*

Target gene	Forward (5’ → 3’)	Reverse (5’ → 3’)
CANX R1	TCGTCGGCAGCGTCAGATGTGTATAAGAGACAGataatcctttttgtttttgaagGATCAGTTCC	GACGTGTGCTCTTCCGATCTGGGTTTTTGTGTCGGAAGATGA
CANX R2	TCGTCGGCAGCGTCAGATGTGTATAAGAGACAGtatgtatttaacacagGTTACTTACAAAGCTCC	GACGTGTGCTCTTCCGATCTtctaacatatatttctctggaaaacacttacC
CALR 1st	GCCGGAGGGTCGTTTTAAAGG	Gggacggtagtgaggtcacc
CALR 2nd	TCGTCGGCAGCGTCAGATGTGTATAAGAGACAGTATCCGTGCCGCTGCTG	GACGTGTGCTCTTCCGATCTgtacagacaaggacttctgcc

1. Two sites for *CANX* were amplified using two sets of primers (R1 for site 1 & R2 for site 2)
2. Both *CALR* editing sites were proximal. However, due to high local GC content two consecutive PCRs were performed to amplify the desired region. In the first (1st), short primers were used to amplify a larger PCR product before a second PCR (2nd) with primers containing Illumina handles were used to focus on the desired region.

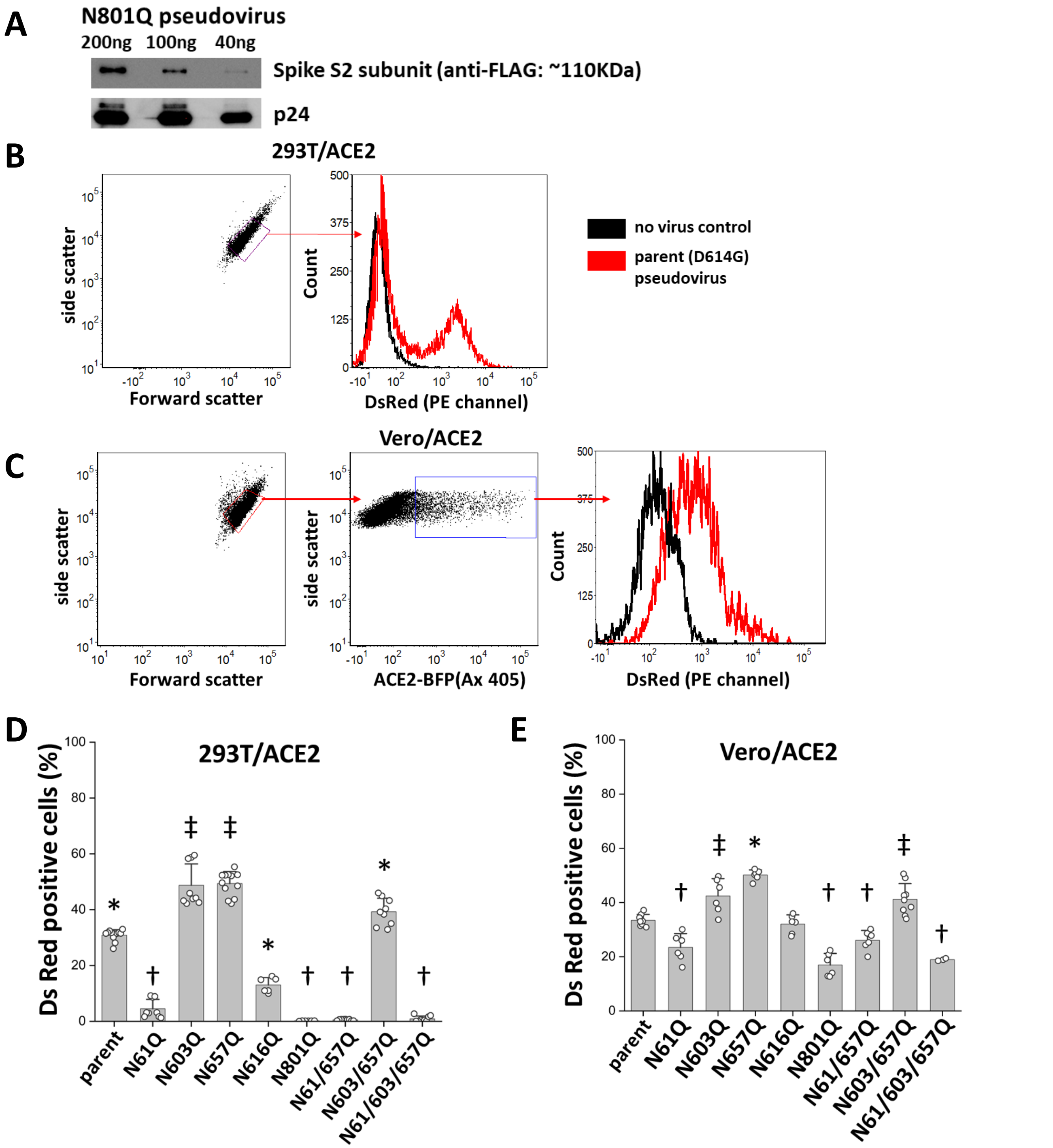

Supplemental Figure S1 (related to Fig. 1) Viral infectivity with Spike mutants. A. Different p24 capsid protein equivalents of N801Q pseudovirus were loaded in lanes. Some Spike can be seen if p24 protein conc. is increased. Thus, the N-glycan mutation reduces, but does not abolish, Spike incorporation into virions. **B-C.** Forward-side scatter was used to identify single cells. Pseudovirus infection results in DsRed signal in host cells. Thus, both % positive DsRed cells and DsRed mean fluorescence intensity (MFI) were measured. This was done both for 293T cells stably expressing ACE2 (‘293T/ACE2, panel B), and Vero E6 cells transiently over-expressing human ACE2 (‘Vero/ACE2’, panel C). In the latter case, ACE2 expressing cells were BFP+, and these were sub-gated prior to DsRed quantitation. Similar to previous reports, Vero/ACE2 displayed less viral entry compared to 293T/ACE2, as indicated by smaller DsRed shift. **D**-**E**. % DsRed positive cells following pseudovirus infection at 72h, measured for both 293T/ACE2 (panel D) and Vero/ACE2 (panel E) cells. The data are consistent with Fig. 1 (main manuscript). However, due to lower viral infectivity in Vero/ACE2 and less distinct DsRed shift (compared to 293T/ACE2), differences are less obvious in panel E. Thus, MFI is a superior measure of viral entry. Data are presented as Mean ± S.D. for N≥3. **P*<0.05 with respect to all other treatments; † *P*<0.05 with respect to ‘parent’; ‡ *P*<0.05 with respect to all other treatments except bars marked by ‡ are not different from each other.

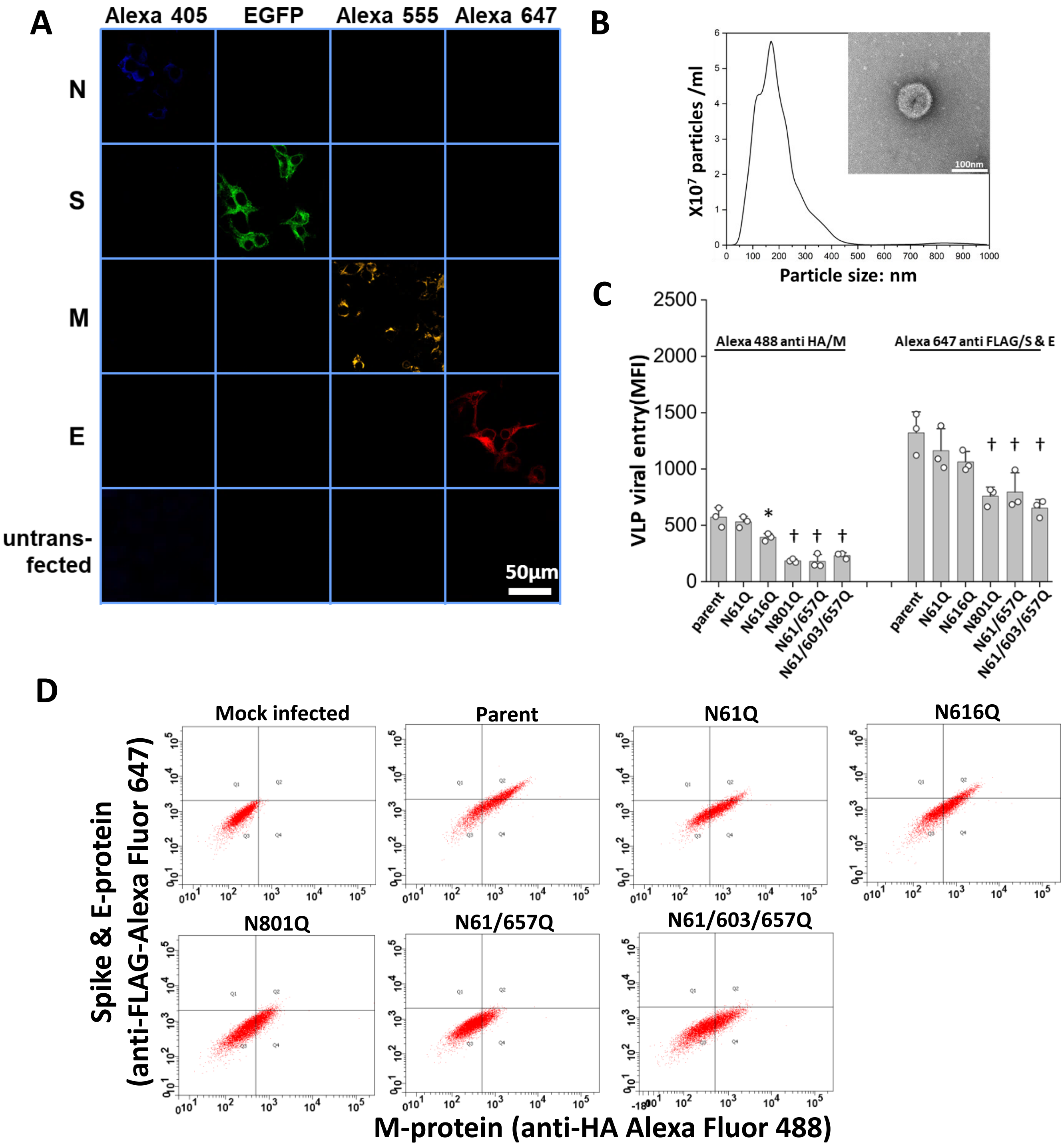

Supplemental Figure S2 (Related to Fig 2). Studies with VLPs. A. Cells were singly transfected with one of the four SARS-CoV-2 structural proteins (E, Spike-EGFP, M or N) or they were untransfected. All fluorescent mAbs, Alexa 405 conjugated anti-Myc (for N-protein), Alexa 555 conjugated-anti-HA (for M-protein) and Alexa 647 conjugated anti-FLAG (for E-protein) were mixed and applied to all fixed-permeabilized cell samples. The images in panel A serve as controls for data in main manuscript, demonstrating the specificity of the detection mAbs and lack of signal leakage between different fluorescence channels. **B.** VLP formed upon co-expression of all four structural proteins was 100-160 nm in size, based on nanosight and negative-stain electron micrographs. **C.** VLPs made using parent Spike and various mutants (no VLP in mock infection control) were spinoculated into 293T/ACE2 cells. Fixed-permeabilized cells were stained with Alexa 647 conjugated anti-FLAG (to detect Spike and E-protein) and Alexa 488 conjugated Anti-HA (to detect M-protein). This enabled quantification of VLP entry. **D.** Representative cytometry plots for data in panel C.

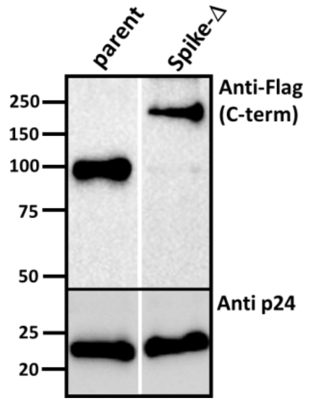

Supplemental Figure S3 (related to Fig. 3). Spike-Δ characterization. Western blot of Spike (parent) and Spike-Δ incorporated into virions, measured using anti-FLAG mAb. Both proteins are expressed equally well in virus. Molecular mass of Spike-Δ is higher (~210kDa) due to lack of S1-S2 cleavage (which would result in a ~90kDa S2-band).

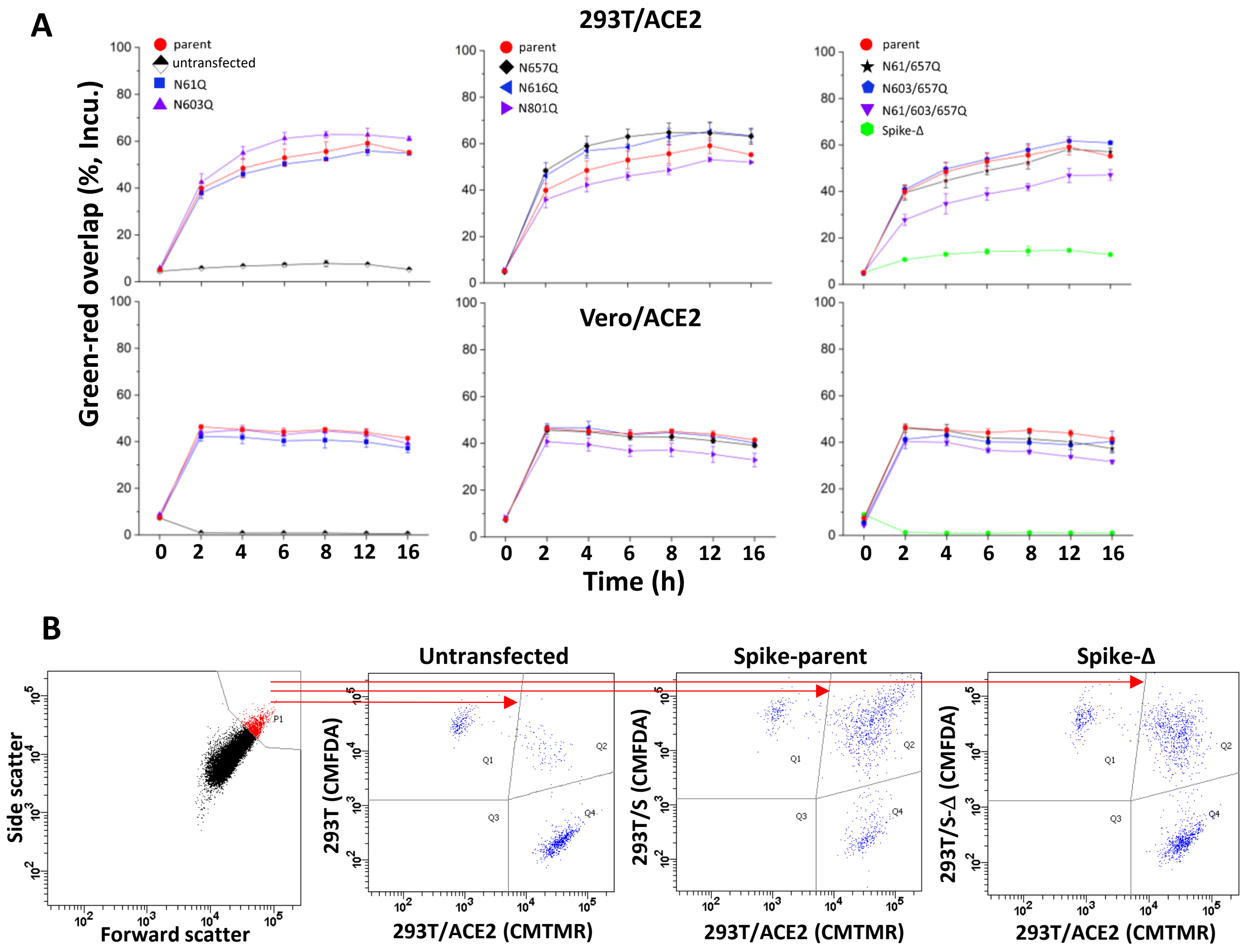

Supplemental Figure S4 (related to Fig. 4) Syncytia formation kinetics. A. Time course of syncytia formation using a mixture of 293T/Spike with 293T/ACE2 cells (top row) or Vero/ACE2 cells (bottom row). Data are Mean $\pm$ S.D. both for parent and mutant Spike constructs (N=3). **B.** Flow cytometry method to measure cell binding plus syncytia formation. Doublets were gated based on forward-side scatter. Independent runs using wild-type 293T and Vero E6 singlets along with FSC-A/-H analysis was used to determine single cell scatter profile. A total of 10,000 cell events were acquired/sampled, with double cells in each quadrant being counted. % association was determined using dual color events in upper-right quadrant (Q2): % association=(# events in Q2/10,000)$\times$100. This quadrant includes cells forming heterotypic aggregates between Spike and ACE2 bearing cells and those forming syncytia. Representative data are presented for 293T/S- 293T/ACE2 mixtures, for: i) untransfected cells without Spike (left panel), ii) Spike-parent (middle) and iii) Spike-Δ (right).

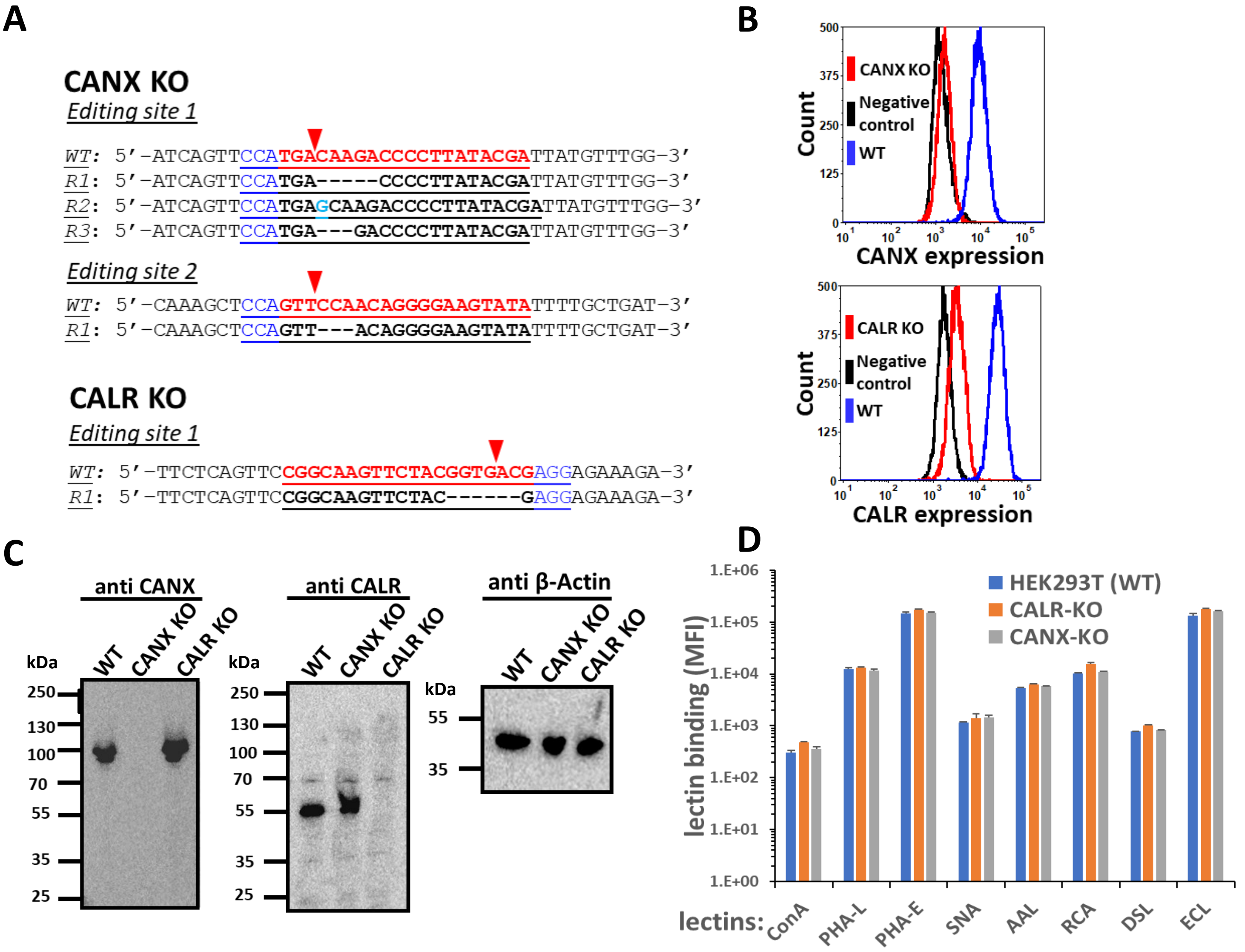

Lectin	Binding specificity	Lectin	Binding specificity
ConA	α(Man) in high mannose	SNA	Neu5Ac(α2-6) linked glycans
PHA-L	Gal(β1-4)GlcNAc(β1-2/6)Man complex	AAL	Fuc(α1-3/6) and also Fuc(α1-2)
PHA-E	Gal(β1-4)GlcNAc(β1-2)Man with bisect	ECL	Gal, lactose based glycans
DSL	GlcNAcβ(1-4/6) linked	RCA-I	Gal, lactose

Supplemental Figure S5 (Related to Fig 5). CANX/CALR knockout cell characterization. A. 293T isogenic clones lacking *CANX* and *CALR* were generated using CRISPR-Cas9. NGS was performed to verify gene editing. Three different indels occurring at ratio 1:1:1 were observed at the first target site of *CANX*, and one edit-type was detected at site 2. A unique indel was detected on *CALR*. While short-read sequencing did not allow characterization of large indels with size >30-35 bases, it did confirm the absence of wild-type sequence in the knockouts. Arrow shows site of frame-shift, 3-4 bases from the protospacer adjacent motif (PAM, blue text). guide-RNA sequence is shown in red. **B.** Flow cytometry analysis of fixed-permeabilized cells confirmed absence of intracellular *CANX/CALR* in the KOs. **C.** Western blot analysis of *CANX/CALR*-KO cell lysates also confirmed complete loss of *CANX/CALR* in these cells. β-actin served as gel loading control. Knocking out one gene did not impact the expression of the other. **D.** Glycan distribution on WT 293T, *CALR*-KO and *CANX*-KO cells was similar, based on flow cytometry assessment and the binding of a panel of fluorescent-lectins. Lectin binding specificity is provided in table form.

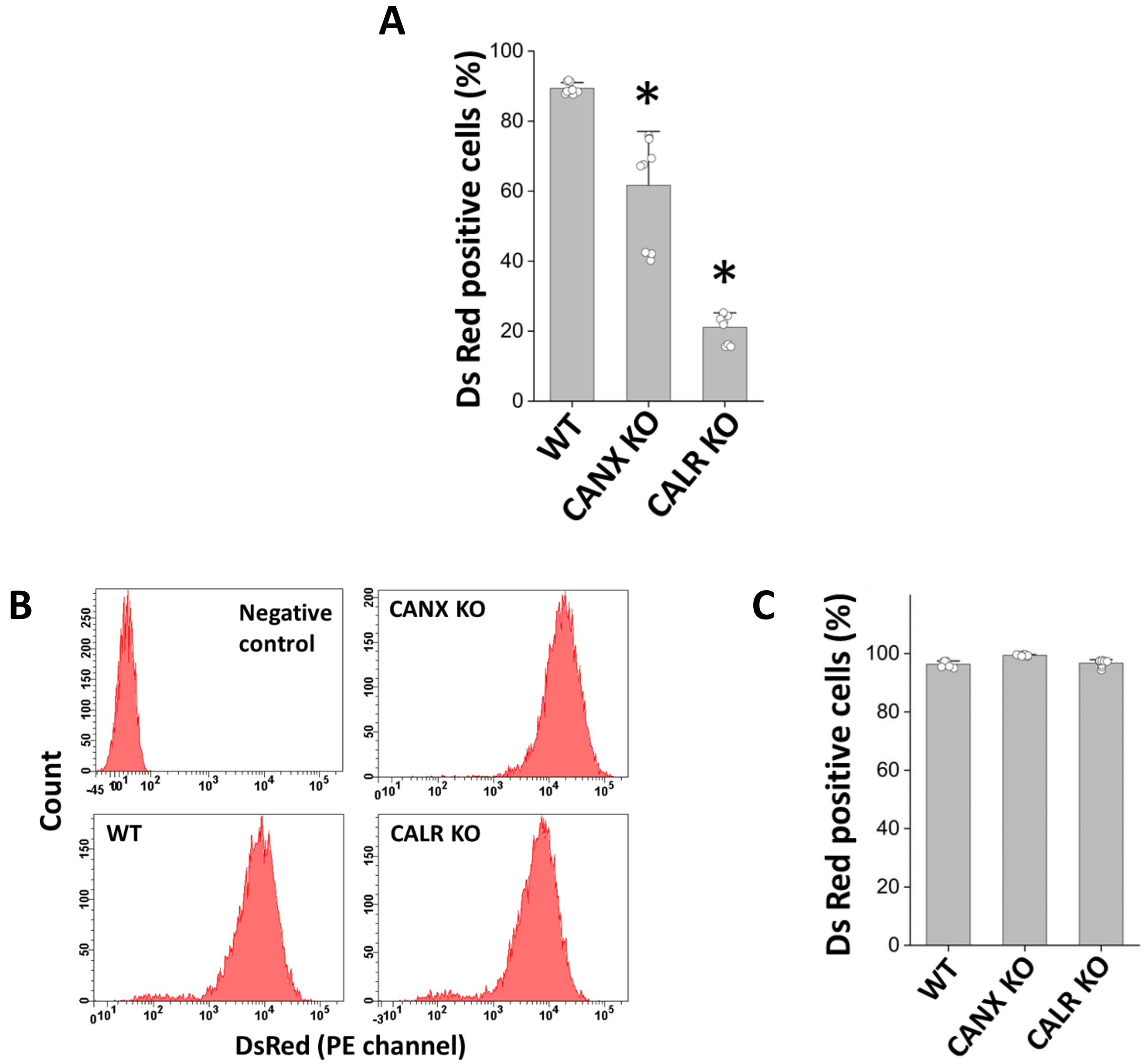

Supplemental Figure S6 (Related to Fig 5). CANX/CALR knockout pseudovirus entry data. A. Pseudovirus bearing 'parent Spike' were produced in WT, *CANX*-KO or *CALR*-KO cells. Viral entry into 293T/ACE2 cells was quantified based on % DsRed positive cells. **B-C.** Same experiment as in panel A, only the envelope protein was VSV-G, instead of 'parent-Spike'. Viral infectivity data are presented using representative cytometry histograms (panel B) and based on quantification of % DsRed positive cells (panel C). Overall, CANX/*CALR*-KO reduced SARS-CoV-2 Spike, but not VSV-G, pseudotyped viral entry. * $P<0.05$ with respect to all other treatments.
